# Leafy Green Farm-to-Customer Process Model Predicts Product Testing Is Most Effective at Detecting Contamination When Conducted Early in the System before Effective Interventions

**DOI:** 10.1128/aem.00347-23

**Published:** 2023-04-26

**Authors:** Gustavo A. Reyes, Jorge Quintanilla Portillo, Matthew J. Stasiewicz

**Affiliations:** a Department of Food Science and Human Nutrition, University of Illinois Urbana-Champaign, Urbana, Illinois, USA; University of Helsinki

**Keywords:** food safety, product testing, simulation

## Abstract

Commercial leafy green supply chains often are required to have test and reject (sampling) plans for specific microbial adulterants at primary production or finished product packing for market access. To better understand the impact of this type of sampling, this study simulated the effect of sampling (from preharvest to consumer) and processing interventions (such as produce wash with antimicrobial chemistry) on the microbial adulterant load reaching the system endpoint (customer). This study simulated seven leafy green systems, an optimal system (all interventions), a suboptimal system (no interventions), and five systems where single interventions were removed to represent single process failures, resulting in 147 total scenarios. The all-interventions scenario resulted in a 3.4 log reduction (95% confidence interval [CI], 3.3 to 3.6) of the total adulterant cells that reached the system endpoint (endpoint TACs). The most effective single interventions were washing, prewashing, and preharvest holding, 1.3 (95% CI, 1.2 to 1.5), 1.3 (95% CI, 1.2 to 1.4), and 0.80 (95% CI, 0.73 to 0.90) log reduction to endpoint TACs, respectively. The factor sensitivity analysis suggests that sampling plans that happen before effective processing interventions (preharvest, harvest, and receiving) were most effective at reducing endpoint TACs, ranging between 0.05 and 0.66 log additional reduction compared to systems with no sampling. In contrast, sampling postprocessing (finished product) did not provide meaningful additional reductions to the endpoint TACs (0 to 0.04 log reduction). The model suggests that sampling used to detect contamination was most effective earlier in the system before effective interventions. Effective interventions reduce undetected contamination levels and prevalence, reducing a sampling plan’s ability to detect contamination.

**IMPORTANCE** This study addresses the industry and academic need to understand the effect of test-and-reject sampling within a farm-to-customer food safety system. The model developed looks at product sampling beyond the preharvest stage by assessing sampling at multiple stages. This study shows that individual interventions and combined interventions substantially reduce the total adulterant cells reaching the system endpoint. When effective interventions occur during processing, sampling at earlier stages (preharvest, harvest, receiving) has more power to detect incoming contamination than postprocessing sampling, as prevalence and contamination levels are lower. This study reiterates that effective food safety interventions are crucial for food safety. When product sampling is used to test and reject a lot as a preventive control, it may detect critically high incoming contamination. However, if contamination levels and prevalence are low, typical sampling plans will fail to detect contamination.

## INTRODUCTION

Leafy greens are an increasingly important commodity in the United States (1). Unfortunately, foodborne disease outbreaks associated with the consumption of leafy greens have increased (2, 3). In the United States, from 2018 to 2020, 3 foodborne outbreaks have been linked to spinach and lettuce contaminated with Escherichia coli O157:H7 (4–6). These repeated outbreaks have caused concern among producers, packers, buyers, and consumers, causing them to manage risk through multiple pathways, including food safety interventions and test-and-reject (sampling) plans. Now as part of the Shiga toxin-producing E. coli (STEC) action plan, the FDA has expressed the importance of prevention methods, especially as new research has identified seasonal effects linked to previous E. coli outbreaks (7, 8).

Contamination of produce can occur at multiple stages of the farm-to-facility process (9). In the preharvest stage, produce may be contaminated through contaminated soil, manure, wildlife intrusion, dust particles, water used for irrigation, and other field activities (10–13). In the harvest and postharvest stages, contamination may occur through direct human contact, cross-contamination from previously contaminated equipment, or other activities (14, 15). Food safety interventions must be considered to manage the residual risk and provide consumers with relatively safe food (16).

To mitigate risk, pre- and postharvest interventions are required as part of regulations and standards (14, 17). Preharvest food safety interventions include controlling adjacent hazards by limiting proximity to animal-rearing operations and urban areas, reducing animal activity through physical barriers, improving worker and equipment hygiene, proper aging controls for manure and soil amendments, and adequate control of water source and irrigation systems. Postharvest interventions include proper handling and temperature controls, product inspection at receiving, and incorporation of a chlorinated wash after cutting (8, 14).

The literature has identified process control verification testing (frequent samples over time) as the most desirable testing to be conducted for many foods, as it demonstrates that a system’s preventive controls are working as intended and minimizes the cost of testing programs (18). However, more traditional lot testing programs such as “hold and release” testing for pathogens at the preharvest or finished product stage are often required by importers and buyers. An example of this is the Canadian Food Inspection Agency (CFIA) requiring romaine lettuce imported from the United States to be sampled and tested for the absence of E. coli O157:H7 (19). Another example may be to comply with product specifications set by buyers, which may require the product to be sampled and tested at different stages to meet acceptance criteria (20). However, the detection of pathogens by product sampling is challenging when prevalence and or levels are low, as in the case of leafy greens (21, 22). The power of sampling is dependent on the total mass collected, the number of grabs, and contamination patterns (23, 24). Validated simulations have shown that many preharvest sampling plans do not reliably detect low-level or low-prevalence contamination (23, 24). Processing typically reduces levels due to the application of interventions, such as washing, spray washing, and preharvest holding time, but the impact of those on sampling power has not been thoroughly studied in leafy greens. Duffy and Schaffner evaluated the general effect of interventions, analyzing microbial sampling pre- and postprocessing for a mock liquid product. The study showed that sampling at the raw material stage was more useful than sampling at the finished product stage due to lower contamination levels postprocessing (25). The leafy green industry would greatly benefit from a study that evaluates sampling’s ability to detect a high-level contamination event at multiple process stages (preharvest to finished product packing).

**FIG 1:**
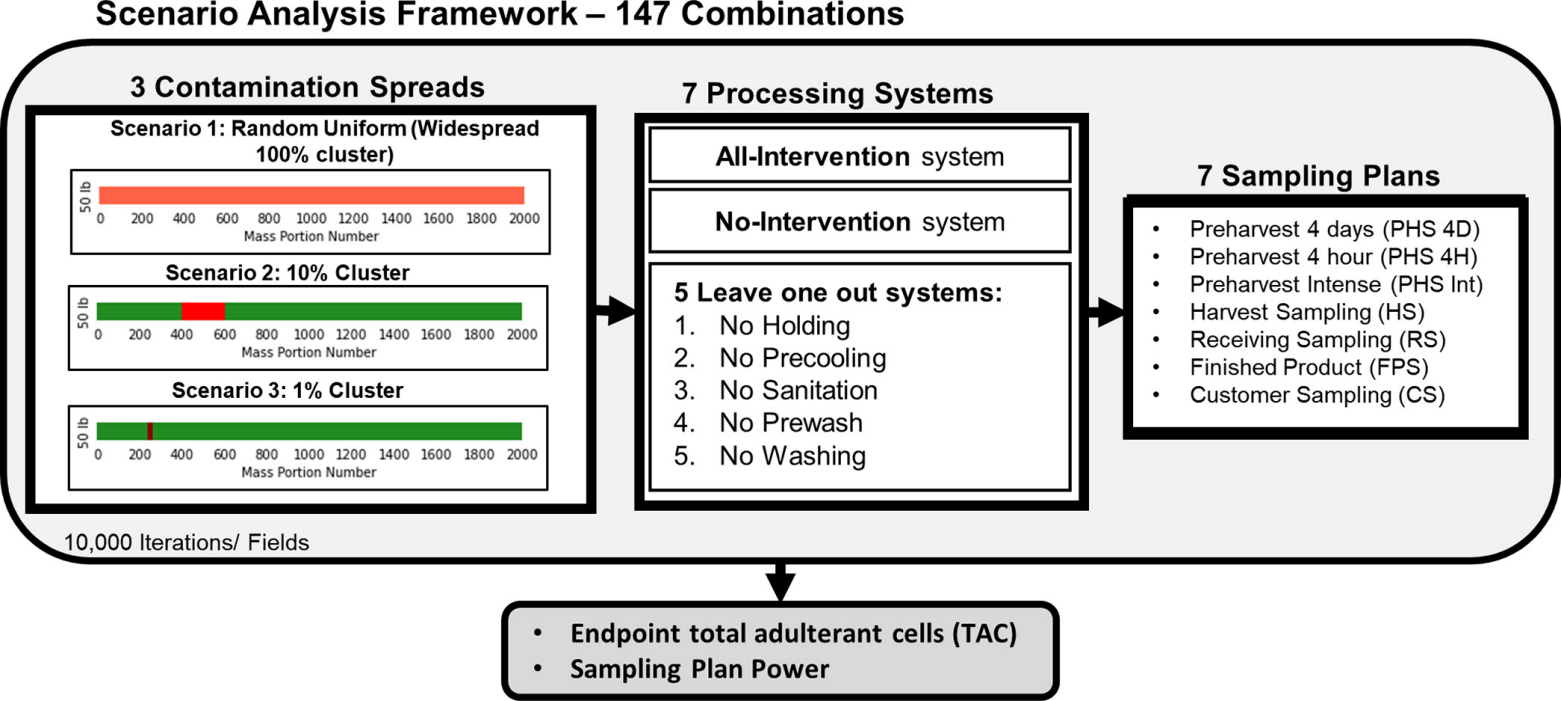
The scope of the scenarios included. Three contamination patterns, seven systems, and seven sampling plans. Each was evaluated individually to predict the sampling plan power and the effect on total adulterant cells (TACs) that could reach the system endpoint.

This study aims to assess the effect of product food safety sampling at different stages of the farm-to-customer process on the total adulterant cells (TACs) reaching the endpoint of the farm-to-customer system in the context of systems with different food safety interventions. Three contamination spreads were modeled to cover the wide range of uncertainty around this parameter: (i) random uniform (widespread 100% cluster), (ii) a large cluster (10% coverage), and (iii) a small cluster (1% coverage). The literature shows that the spread of preharvest contamination is uncertain, ranging from widespread contamination to small clusters of contamination depending on the contamination source (10, 26–28). Seven systems were modeled to represent different levels of adherence to food safety practices. (i) An all-intervention system (preharvest holding, precooling, prewash, wash, and processing line sanitation), (ii) a no-intervention system (a system without those five interventions), and (iii) five other in-between systems where each one of the five food safety interventions fails. The model implements sampling at seven process stages from preharvest to customer. The combinations of all these treatments resulted in 147 combinations (Fig. 1). As a measure of food safety management, these combinations will be compared to determine the effect of sampling at different stages and under different systems on the endpoint TACs.

## RESULTS

### Individual interventions reduced contamination levels throughout the system, and combined effective interventions achieved a greater reduction.

**FIG 2:**
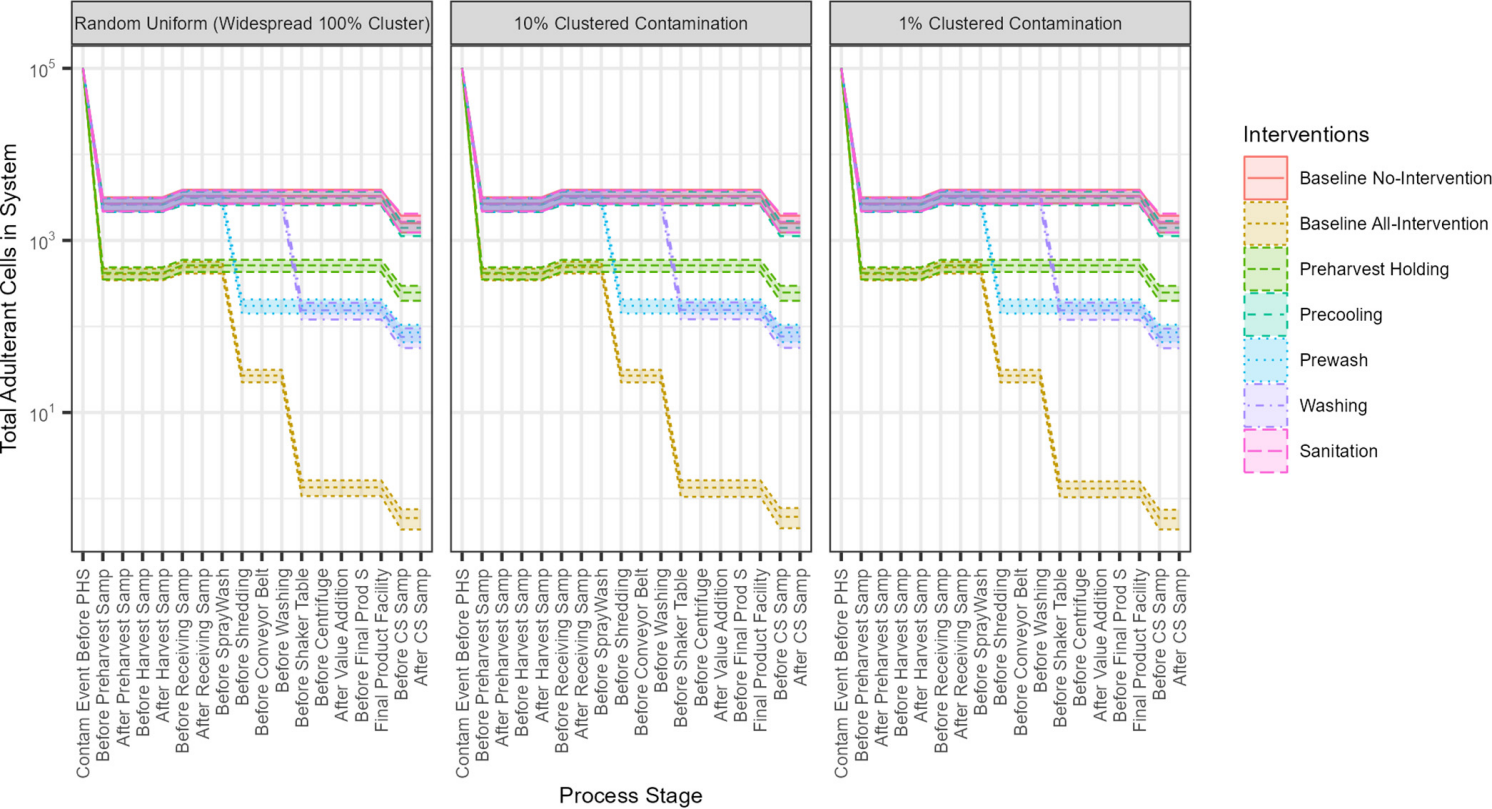
Contamination progression. Each panel represents a contamination scenario. The *x* axis represents system steps, and the *y* axis represents the total adulterant cells (TAC) present in a system. The yellow bar is the all-interventions system, and the red bar is the no-interventions system. The other colored lines represent different systems with different individual interventions. The lines represent the mean, and the shaded areas represent the 95% CI of the mean. A drop in the TACs compared to the no-intervention system demonstrates the effect of each or all interventions.

The main objective of this model was to determine the effect of sampling at different stages of the farm-to-customer system in the context of the system with other intervention strategies. To achieve that, we first studied the effect of five interventions: (i) preharvest holding, (ii) precooling at receiving, (iii) prewash, (iv) chlorinated wash, and (v) processing line sanitation. Contamination in the system, total adulterant cells (TACs), and progression were tracked for the all-intervention system, the no-intervention system, and the systems with single interventions (Fig. 2).

When all interventions were applied to the system, the total reduction of adulterant cells at the endpoint compared to no interventions averaged 3.43 log TACs (95% confidence interval [CI], 3.33 to 3.56). The two most effective interventions were the chlorinated wash and the prewash, which reduced the final TACs in the system by 1.32 log TACs (95% CI, 1.22 to 1.45) and 1.27 log TACs (95% CI, 1.18 to 1.38), respectively. The third most effective intervention was the implementation of holding time at preharvest, providing an average 0.80 log TACs (95% CI, 0.73 to 0.90) reduction. Interventions that had minor effects were conducting sanitation of the processing lines and precooling, providing an average reduction of 0.06 log TACs (95% CI, 0.02 to 0.15) and 0.01 log TACs (95% CI, −0.10 to 0.11), respectively. The poor efficacy of sanitation was due to the transfer coefficients between produce and surfaces being low, as well as contamination during processing being relatively low; therefore, contamination did not accumulate on the processing surfaces.

### The power of sampling plans depends on the contamination levels at each sampling point.

Sampling plan power describes the ability of a given sampling plan to detect contamination at a given system stage under the sampling conditions stated in Table 1. Sampling plan power refers to the number of iterations where the sampling plan was able to detect contamination over the total number of iterations (*n* = 10,000). Higher power means the sampling plan is more likely to detect contamination at a given system stage.

**TABLE 1:** Description of sampling plans for each sampling scenario

Sampling plan	Total no. of samples	Subsample description	Total mass sampled (g)	Subsample mass (g)	Grabs per subsample (*n*)	Total grabs (*n*)	Wt per grab (g)	Rejection rule
Preharvest 4 days (PHS 4D)	1	4, 375 g	1,500	375	15	60	25	Total mass
Preharvest 4 h (PHS 4H)	1	4, 375 g	1,500	375	15	60	25	Total mass
Preharvest sampling intense (PHS Int)	4	4, 375 g	6,000	375	15	240	25	Total mass
Harvest sampling (HS)	1	4, 375 g	1,500	375	15	60	25	Total mass
Receiving sampling (RS)	1	1, 375 g every 6–7 pallets	1,500	375	15	60	25	Total mass
Finished product sampling (FPS^*a*^)	1	1 subsample every 3.5 h of production	1,500	375	15	60	25	Total mass
End consumer sampling (CS)	1	1, 375 g every 15–16 pallets	1,500	375	15	60	25	Total mass

a Based on the day being 14 h of production as per reference 38.

**FIG 3:**
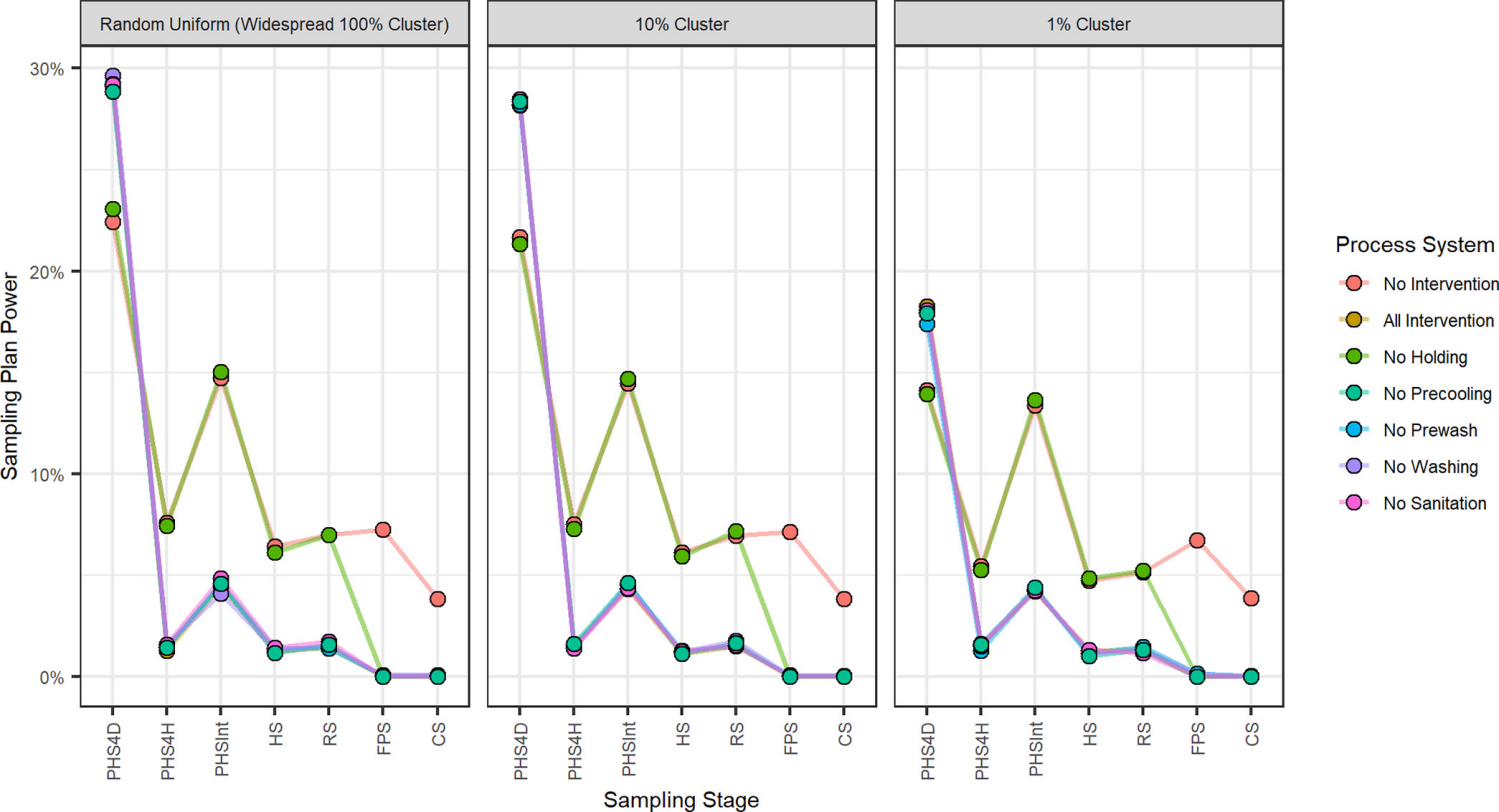
Sampling plan powers stratified by clustering levels and system. Sampling plan power is defined as the total number of iterations where the sampling plan detected the contamination/total number of iterations (*n* = 10,000) The results indicate that preharvest sampling 4 days (PHS 4D) was the most powerful sampling plan, with power ranging between 14.1% and 29.6%. Sampling plans at later stages, such as finished product sampling (FPS) and customer sampling (CS), show lower sampling plan power, especially when interventions are implemented.

The power to detect contamination of the 7 sampling plans (Table 1) at the 7 sampling stages and 3 contamination scenarios (147 total combinations) is summarized in Fig. 3 (see also Table S2 in the supplemental material). First, sampling plan power was lower for those sampling plans that occurred later in the system; this was due to contamination at sampling points being lower as the system progressed, due to natural die-off through the system or reduction due to effective interventions. Second, except for preharvest sampling 4 days before harvest (PHS 4D), effective interventions reduced the power of sampling plans; this was because the application of effective interventions resulted in lower TAC contamination at each sampling point. Third, the degree of clustering matters; highly clustered contamination (e.g., 1% cluster) is harder to detect (lower power) earlier in the system.

The most effective sampling plan across all systems was the preharvest sampling for 4 days (PHS 4D), with power ranging between 14.1% and 29.6%. Opposite to all the other sampling plans, the power of the PHS 4D sampling plan increased when an intervention was in place, in this case, the preharvest holding intervention. This intervention limited the contamination window to 2 to 8 days before harvest, compared to the 0 to 8 days when it was not in place. With a narrower contamination window, the probability of a contamination event occurring before PHS 4D was higher, as well as contamination at the sampling point being higher, and hence the higher sampling plan power. The disadvantage of this sampling plan was that contamination could still happen after sampling occurred, since sampling occurred 4 days before harvest, causing the plan to potentially miss a contamination event between PHS 4D and harvest. Contamination levels at PHS 4D for the systems that applied holding as an intervention were, on average, between 17,431 and 17,447 TACs, whereas for the systems that did not apply holding as an intervention (no-intervention and no holding), the contamination levels were lower, 12,983 (585 to 69,661, 90% variability interval) and 13,024 (587 to 70,032, 90% variability interval) TACs, respectively. Demonstrating that for this step, if the holding intervention were to fail, sampling would have reduced power for informing a contamination event.

Preharvest sampling 4 h (PHS 4H), preharvest sampling intense (PHS Int), and harvest sampling (HS) were sampling plans that were solely affected by the preharvest holding intervention. These three sampling plans showed lower sampling plan power when the holding intervention was in place, meaning that the all-intervention, no-washing, no-prewash, no-precooling, and no-sanitation systems had lower sampling plan power than the no-intervention and no-holding systems at these three sampling stages. TAC levels at each sampling point were lower when holding was in place, on average, between 401 and 503 TACs for PHS 4H, PHS Int, and HS. When holding was not in place (no-intervention and no-holding systems), the contamination levels were meaningfully higher, between 2,600 to 3,360 TACs for PHS 4H, PHS Int, and HS, demonstrating that (i) sampling plan power depends on the contamination levels at the sampling point and (ii) effective interventions reduce contamination, therefore, making the remaining contamination harder to detect. (iii) If the holding intervention were to fail, sampling would provide more information than when this intervention is working as intended.

Receiving sampling (RS), in addition to being affected by holding, was also affected by the precooling intervention. When holding or precooling interventions were not in place, TACs at RS were, on average, between 484 to 490 TACs. TACs were lower compared to when there was no precooling (492 TACs), when there was no holding (3,170 TACs), or when no interventions were in place (3,205 TACs). This demonstrates that if the precooling system were to fail, sampling would not provide much additional information about the failure of this intervention. In contrast, if the holding intervention were to fail, sampling would have a higher chance of detecting the remaining contamination.

Finished product sampling (FPS) and customer sampling (CS) could be affected by all five interventions. These two sampling plans followed the same pattern as the other sampling plans. When effective interventions failed, contamination levels were higher at the sampling point, making it easier for sampling plans to detect contamination events. When the most effective intervention, washing, was not implemented in the system, TACs were, on average, 13 to 27 at FPS and CS. When holding was not in place, TACs, on average, were 4 to 9. When all interventions were in place, TACs were, on average, 1 for both FPS and CS. These results show that even when effective interventions fail, contamination levels remained relatively low because of other interventions. When no interventions were applied, endpoint TACs were, on average, between 1,550 and 3,200 at FPS and CS.

Figure 3 shows that sampling plan power remained close to 0% for all systems when one intervention failed. This means the small differences in contamination when one intervention fails have small effects on sampling plan power. Sampling might still be able to detect a complete system failure, as seen by the higher power in the no-intervention system.

### The efficacy of sampling plans is dependent on interventions and system stage.

The endpoint TACs were additionally used to compare two patterns: (i) the relative efficacy in endpoint TACs between sampling plans for each of the 7 systems and (ii) the log_10_ difference and the relative efficacy of each system (losing an intervention) on endpoint TACs compared to the all-intervention system (Table 2). These two patterns allowed us to compare the relative effect that product sampling and removing one intervention at a time had on the endpoint TACs.

**TABLE 2:** Endpoint total adulterant cells (TAC) comparison between the No-Intervention scenarios and the All-Intervention scenarios. Log_10_ change calculated by calculated as log_10_ (All-Intervention/No- Intervention)

Intervention	Random uniform (widespread 100% cluster)			10% cluster			1% cluster		
	Endpoint TACs (*n*)	Log change	Relative efficacy (%)	Endpoint TACs (*n*)	Log change	Relative efficacy (%)	Endpoint TACs (*n*)	Log change	Relative efficacy (%)
No intervention	15,851,671	0.00	0.00	15,841,829	0.00	0.00	15,823,153	0.00	0.00
All intervention	5,939	−3.43	99.96	6,139	−3.41	99.96	5,903	−3.43	99.96
No holding	40,238	−2.60	99.75	40,425	−2.59	99.74	38,815	−2.61	99.75
No precooling	6,171	−3.41	99.96	6,482	−3.39	99.96	6,618	−3.38	99.96
No prewash	105,908	−2.18	99.33	105,674	−2.18	99.33	105,561	−2.18	99.33
No washing	118,506	−2.13	99.25	118,464	−2.13	99.25	118,545	−2.13	99.25
No sanitation	5,606	−3.45	99.96	5,668	−3.45	99.96	5,576	−3.45	99.96

**FIG 4:**
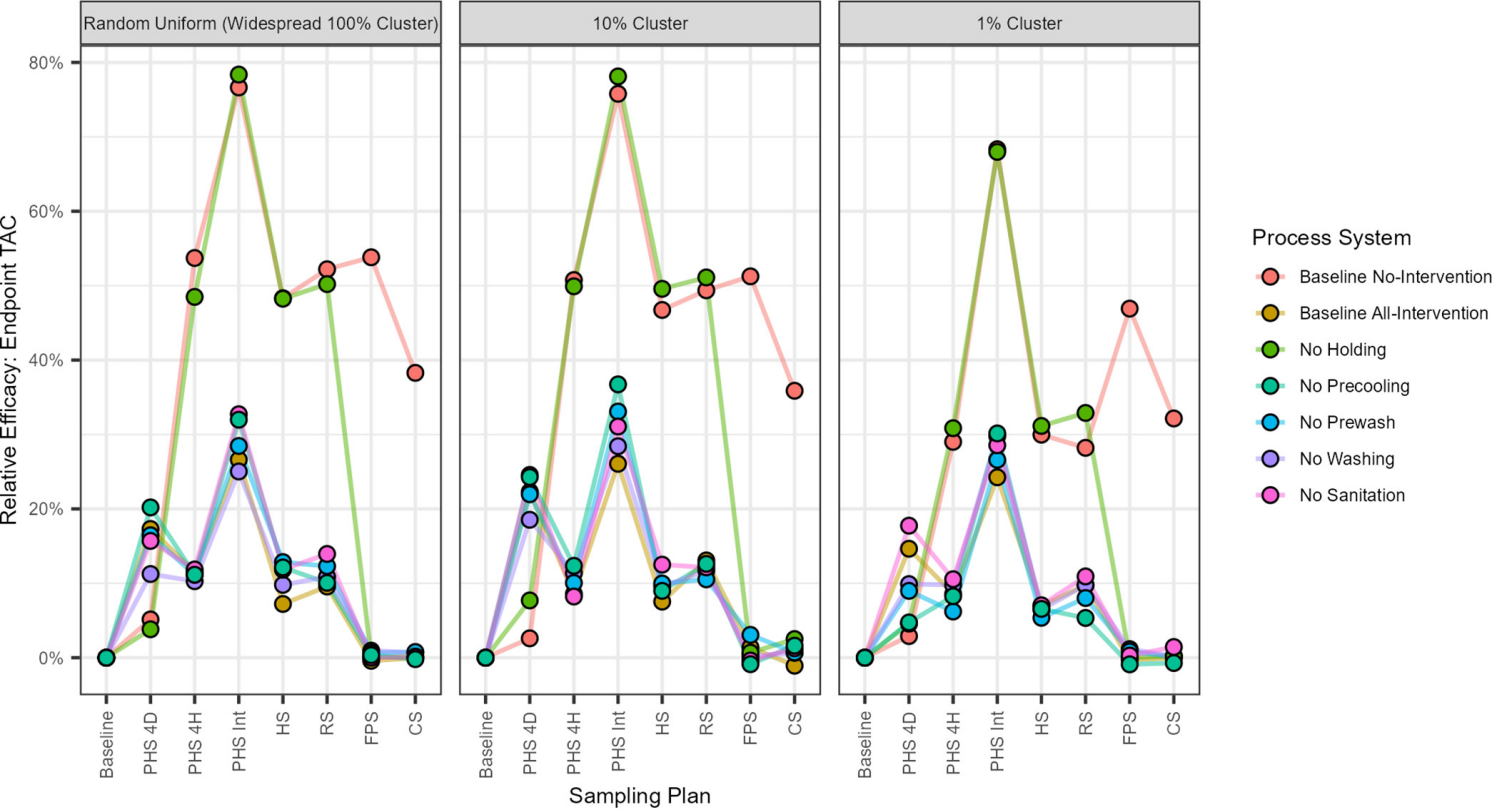
Final relative endpoint total adulterant cells (TAC) from 10,000 simulated contaminated iterations. This represents the relative endpoint TACs compared to each system. A lower relative difference of endpoint TACs means that the sampling plan was more effective than its given processing scenario. The results indicate that the sampling plan that most effectively reduced endpoint TACs is the preharvest intense (PHS Int) sampling plan. Sampling plans were shown to be less effective when effective interventions were in place. Effectiveness increased when no intervention was in place. When effective interventions were in place, sampling at the final stages, such as finished product sampling (FPS) and customer sampling (CS), resulted in the lowest efficacy. When no effective interventions were in place, sampling at the finished product or customer sampling was shown to be more effective.

The same relative efficacy achieved by incorporating each sampling plan was quantified for each of the 147 combinations. Table S3 shows the relative efficacies. A visualization of the 147 combinations is shown in Fig. 4. The main pattern suggests that sampling plans had a lower effect on reducing endpoint TACs when multiple effective interventions or all interventions were in place, except for PHS 4D. PHS 4D had the highest relative effect when the all-intervention system and those systems that included the holding intervention were in place, for reasons explained above. For PHS 4D, the relative efficacy in endpoint TACs ranged between 5% and 25% for systems that included the holding intervention. For those systems that did not include the holding intervention (no intervention and no holding), relative efficacy was lower, ranging between 3% to 8%. This result predicts that PHS 4D alone had at most an 8% relative reduction in the endpoint TACs, compared to the higher relative efficacy achieved (5% to 25%) when it was paired with the holding intervention.

The assessment predicted that the remaining sampling plans had a lower relative effect on reducing endpoint TACs when multiple effective interventions or all interventions were in place for all other sampling plans. The most effective sampling plan across all systems was preharvest sampling intense (PHS Int). PHS Int showed a relative efficacy in endpoint TACs of between 68% and 78% for systems that did not apply the holding intervention, while for those systems affected by the holding intervention, the relative efficacy in endpoint TACs was lower, between 24% and 37%, demonstrating that sampling provides greater relative reduction to the endpoint TACs when no effective interventions or no interventions are in place compared to when effective interventions such as holding are in place. A similar pattern can be observed for other sampling plans later in the system. Finished product sampling (FPS) and customer sampling (CS) may be affected by all 5 interventions. When no interventions were in place, the relative efficacy in endpoint TACs ranged between 47% and 54% for FPS and 32% to 38% for CS. When all interventions were in place, the relative efficacy in endpoint TACs was between 0% and 1%, predicting that sampling at these later stages provided no effect when the optimal system was in place. Similarly, for the other systems, when one intervention at a time failed, FPS and CS did not provide much value; the relative efficacies ranged between 0% and 3%, predicting that for these effective systems, it is most beneficial to conduct sampling earlier in the system, potentially detecting high contamination loads, rather than after multiple reduction steps occur.

The second pattern was to quantify the effect on relative efficacy that removing one intervention at a time had compared to having all and no interventions. A summary of the results can be found in Table 2. The results indicate that even if one intervention fails, the endpoint TACs will still be reduced by at least 99% by the remaining effective interventions. When all interventions are in place, the relative reduction to endpoint TACs increases to 99.2%. While some sampling plans were able to have a relatively meaningful effect on the endpoint TACs, the relative reduction achieved by sampling does not compare to the relative reduction of endpoint TACs achieved by implementing effective interventions, 99.96%. Even when effective interventions such as washing, prewashing, and holding were removed, the relative efficacy of the systems remained high, 99.27%, 99.31%, and 99.75%, respectively. Nevertheless, in all these scenarios, adulterant cells still reached the endpoint of the system, showing that even with effective interventions, the risk is not fully mitigated under these outbreak-like conditions; even the best-simulated system has residual risk.

### Factor sensitivity shows that sampling before processing interventions leads to greater adulterant reductions than sampling after processing interventions.

**FIG 5:**
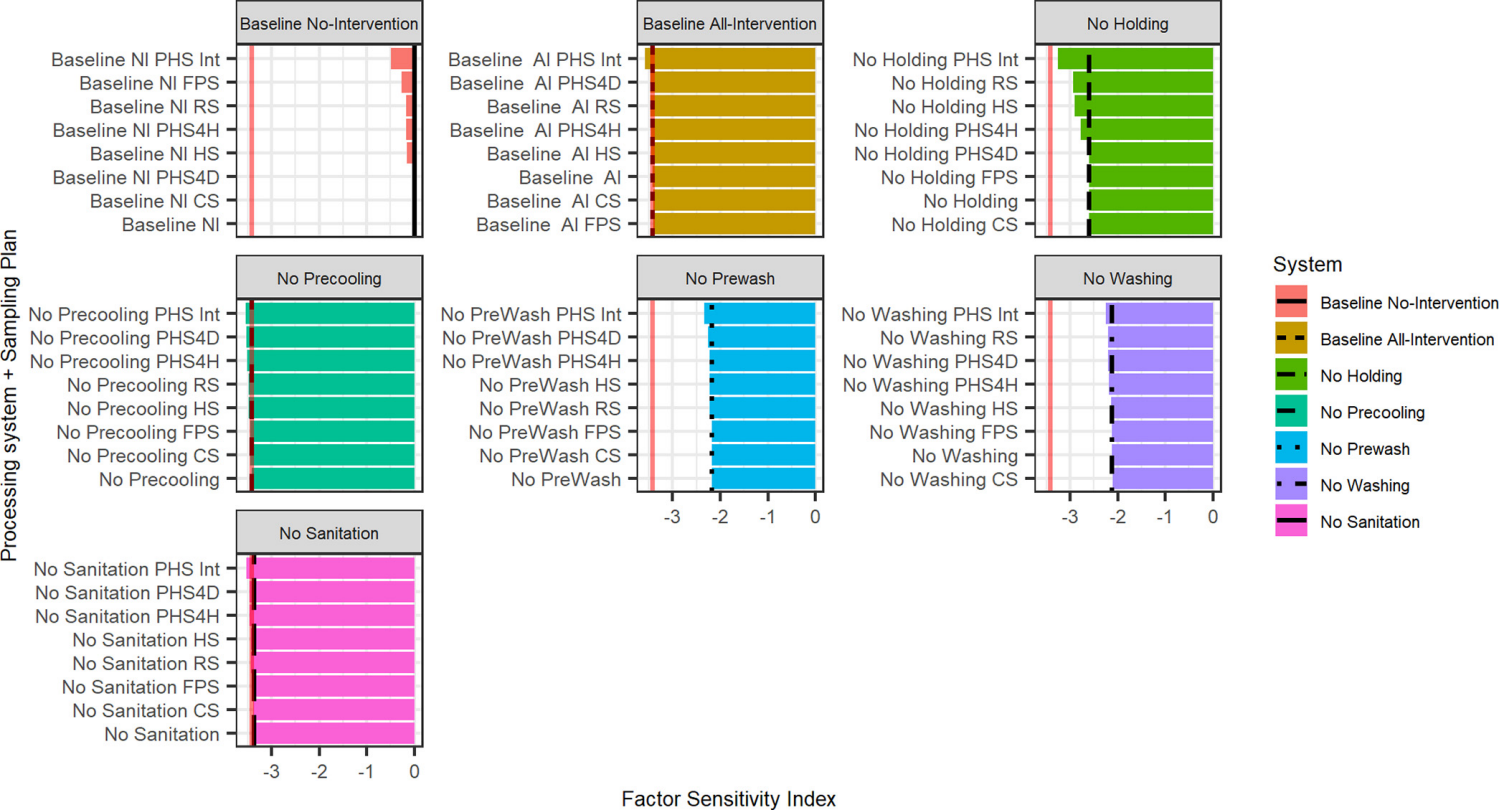
Results of the factor sensitivity analysis. Factor sensitivity (FS) is the log reduction between the endpoint from each scenario and the system with no food safety interventions (log_10_ [intervention/no-intervention system], for 10,000 iterations). The greater the absolute value of the FS, the more effect that specific scenario or condition has on endpoint TACs. The red lines represent the effect of the all-intervention system; the black lines represent the effect of each system without sampling. The difference between the red and black lines represents the efficacy lost by removing interventions. The goal of the sampling plans is to take the bars beyond the black lines; the greater the distance between the black line and the bar, the greater the effect of a sampling plan in that specific system. Interventions have greater factor sensitivity than the sampling plans. When the all-intervention system was in place, sampling plans showed small to no effect.

Factor sensitivity (FS) analysis (Fig. 5) assessed the effect of sampling plans across each of the seven what-if systems. The FS provides information on which sampling plans would most efficiently reduce endpoint TACs if a specific intervention were to fail, as well as on how removing interventions decreases the overall efficacy of the optimal all-intervention system.

The factor sensitivity analysis reiterates and shows very similar results to what was previously shown in Table 2 and Fig. 2. The all-intervention system will have an efficacy reduction when the most effective interventions are removed from the system; this is the distance between the black lines and the red line in Fig. 5. In the FS analysis, removing washing, prewashing, and holding alone decreased the all-intervention system’s efficacy by 1.30, 1.24, and 0.81 logs, respectively. Removing interventions with lower efficacy, such as precooling and sanitation, reduced all intervention efficacy by 0.02, and 0.06 logs. When all interventions were removed from the system the effectiveness was reduced by 3.42 logs.

The most effective sampling plan across all systems was the preharvest intense system (PHS Int), with added reductions between 0.13 and 0.66 log endpoint TACs. This is represented by the distance between the black line and the end of the bar in Fig. 5. For the no-intervention system, the second and third most effective sampling plans were the finished product sampling (FPS) and receiving sampling (RS) systems, with added reduction of 0.26 log and 0.17, respectively. For the all-intervention system, the second and third most effective plans had limited effects, the preharvest sampling 4 days (PHS 4D) and receiving sampling (RS), had an added log reduction of 0.053 and 0.054, respectively.

For the no-sanitation, no-precooling, no-wash, and no-prewash systems, the second and third most effective sampling stages were the preharvest sampling 4 days (PHS 4D) followed by preharvest sampling 4 h (PHS 4H), with reductions of between 0.084 to 0.11 log and 0.054 to 0.092 log, respectively. For the no-holding system, the second and third most effective sampling plans were receiving sampling (RS), and harvest sampling (HS), with reductions of 0.34 and 0.30, respectively.

In addition, for all the systems except for the no-intervention system, the least effective sampling plans were finished product sampling (FPS) and customer sampling (CS), with added reductions ranging between 0 and 0.037, and 0 and 0.031 log, respectively.

**FIG 6:**
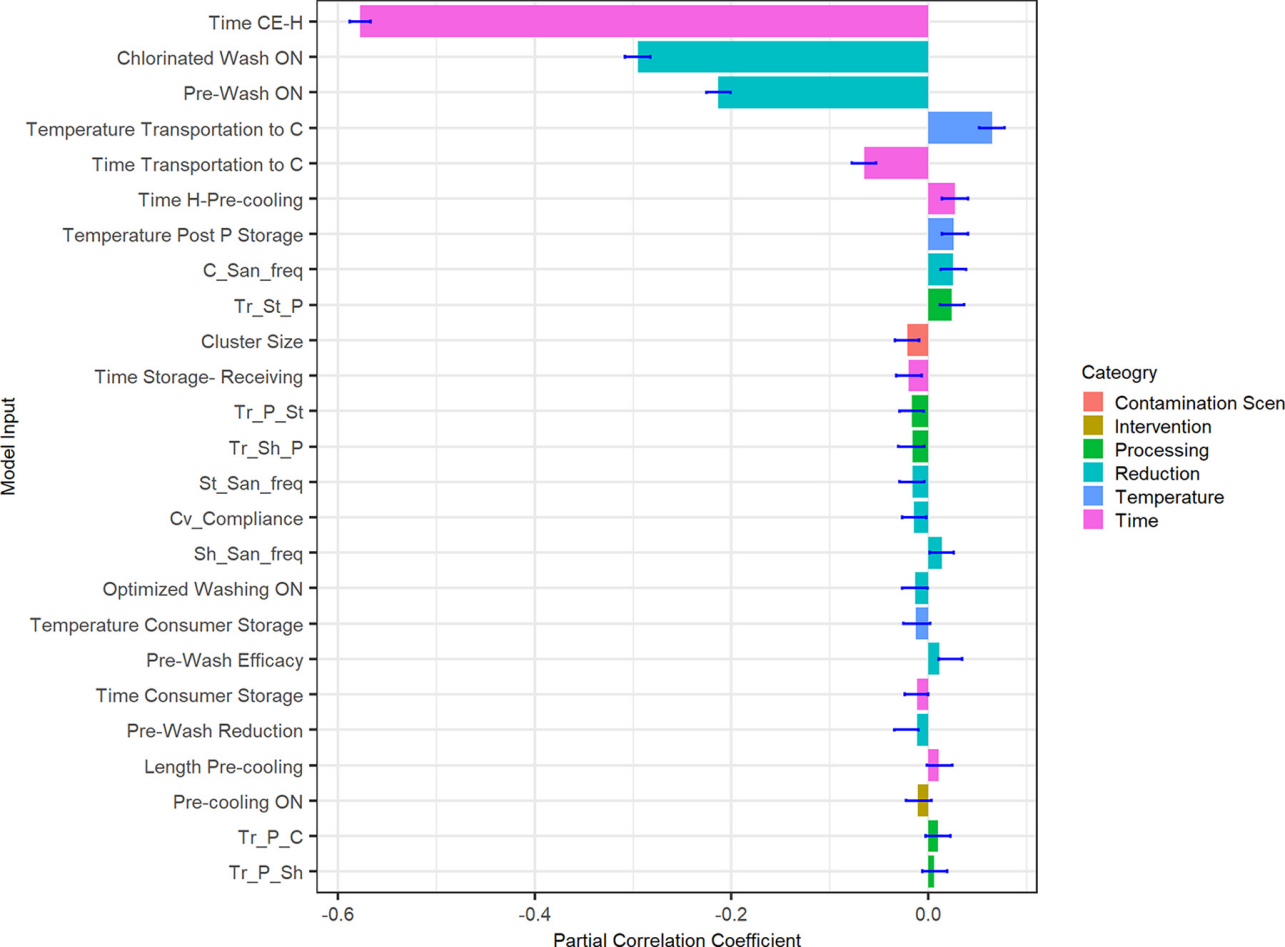
Results of the partial rank correlation sensitivity analysis. The greater absolute partial rank correlation coefficient (PRCC) represents the greater effect of that specific input in the final contamination of adulterant cells at the end of the iteration.

Beyond assessing major system interventions and sampling plans with an FS, a partial rank correlation coefficient (PRCC) sensitivity analysis (Fig. 6) was performed to obtain a complete profile of endpoint TACs of all randomized input variables. The inputs with the largest absolute PRCCs, indicating the most sensitivity in the time between the contamination event and harvest (−0.58), were chlorinated wash on (−0.29), prewash on (−0.21), and transportation time to consumer (−0.06). The PRCC also showed sampling plans to have the smallest PRCC values, ranging between −0.005 and 0.021.

These sensitivity analyses together suggest that the incorporation of effective interventions, such as preharvest holding or process washing, meaningfully reduce endpoint TACs. These also show that when effective interventions are in place, sample testing and rejection before processing result in larger reductions. If no interventions are in place, sample testing and rejection of the finished product lead to larger reductions.

## DISCUSSION

### Process interventions reduce contamination that reaches the system endpoint.

The analysis predicted an overall impact of food safety interventions of around 3.5 log, with individual interventions achieving 0.8- to 1.3-log reductions, a range consistent with other models in the literature that have tracked microbial loads through produce systems (29–31). Washing of leafy greens has been predicted to be a food safety intervention, as also predicted in this model. Pang et al. demonstrated that a chlorinated wash would result in a 12.7-fold reduction of illness (from 2,160 to 170 illness per year in the US) caused by leafy greens compared to the baseline system in their quantitative microbial risk assessment (QMRA) (32). In a different study, Danyluk and Schaffner indicated that washing may not only provide a reduction in contamination but may also reduce the degree of cross-contamination that occurs during the washing process, providing a significant reduction of the E. coli O157:H7 levels on leafy greens (30). Mokhtari et al. showed that compliance and an effective preliminary spray wash are an effective intervention to reduce contamination levels in the finished product (29). A holding time between final irrigation and harvest is another critical strategy to control the bacterial load on harvested produce. A QMRA conducted by Ottoson et al. showed that holding time is extremely important for illness reduction. In that study, the baseline model, 0 days of holding time, resulted in 436 illnesses per 10,000 servings. In contrast, the system with 2 days of holding time reduced the illness prevalence to 52 illnesses per 10,000 servings, a significant reduction (33). These studies highlight the importance of implementing systems such as good agricultural and manufacturing practices (GAPs and GMPs) to control contamination as recommended by the FDA (8).

### Contamination levels at the sampling point and sampling plan characteristics determine sampling plan power.

The power of sampling plans depends on the adulterant cells at the sampling point. Overall samples taken earlier in the system (such as preharvest, harvest, and receiving) were more powerful than those taken after effective processing interventions (such as at finished product sampling and customer sampling). Statistical analysis (34) supports that sampling plan power is higher when contamination levels are higher. Simulation analysis (25) specifically shows that as the contamination level decreases through effective food safety interventions, the power of the sampling plan decreases.

The power of sampling also depends on the sampling plan characteristics (composite sample mass and the number of individual grabs taken). In our scenario analysis, preharvest sampling intense (PHS Int) had a 4-fold increase in the composite sample mass and number of grabs taken compared to harvest sampling (HS), at the same sampling point. Preharvest sampling intense outperformed harvest sampling by 2.5- to 4.0-fold. Therefore, our study agrees with other studies that show that sampling plans with higher sample mass and more sampling grabs perform better in detecting contamination (23, 24, 34).

The sampling plans analyzed in this study would mostly fail to detect a preharvest contamination event with contamination levels of 1 CFU/lb. Other studies have also concluded that typical sampling strategies would fail to detect in-field contamination specifically at low levels and prevalence (13, 25, 27). The total sample mass and the total number of grabs would need to increase to improve the performance of the sampling plans (24). However, increases are constrained by the feasibility of labor and laboratory analysis costs. Our study adds another suggestion to increase power. Sampling before effective process interventions (preharvest, harvest, or receiving) will typically result in a higher power due to higher contamination levels at the sampling point.

### The predicted power of sampling for a test-and-reject program does not fully describe the impact of that sampling program.

Sampling plan power provides information on how well the sampling plan performs for a given sampling stage. However, that metric does not directly addresses how many contaminants reach the customer when sampling is used for the testing and rejection of lots. A sampling plan might have higher power because it detects contamination when the contamination levels are high (preharvest), making detection more likely. But effective food safety interventions later in the system might otherwise reduce that contamination, lowering the relative immediate food safety impact of sampling, where many of the cells detected early would already be managed and reduced, though such detection would still be valuable in the long term for understanding what causes contamination and informing continuous improvement.

For example, the most powerful preharvest sampling in our model (PHS 4D) had similar effects on reducing the contamination that reached the customer compared to other preharvest sampling plans. The sensitivity analysis showed that the most important factor in reducing preharvest contamination levels is the preharvest die-off. Belias et al. (35) showed that contamination at preharvest declined at a variable rate with a mean and standard deviation of −0.77 and 0.21 log(adulterant cells per day), respectively. If the event happens 8 days before harvest, the microbial population might naturally be reduced to very low levels before harvest occurs, limiting the direct food safety impact of preharvest sampling. The model suggests that the most powerful samples would be those taken as close as possible to the contamination event, as shown by Duffy and Schaffner (25).

### The value of testing.

Buchanan and Schaffner describe different types of microbial testing within a food safety system (18). In food systems, the most beneficial and desired type of testing is process control verification testing, as it may be able to detect when preventive measures may be out of control. In addition, the produce industry is often required to conduct hold-and-release testing to comply with import or buyer requirements as a preventive control, to prevent contaminated products from entering the market. While beneficial if contamination is detected, the effectiveness of this type of testing decreases substantially when the prevalence of contamination is low, as many samples would be needed to detect contamination.

Our results show that effective food safety interventions reduce contamination more than the effect of sampling for rejecting contaminated lots. As mentioned above, test-and-reject sampling is powerful when contamination is high, tests identify an adulterant, and rejection effectively reduces the number of cells that would otherwise pass through the system. Here, we show that sampling is typically more powerful early in the system, but then subsequent interventions would otherwise reduce contamination if it were not detected, reducing the impact of testing for rejection. In addition, sampling at later stages after effective interventions take place has lower detection power because of the lower contamination prevalence and levels as an effect of effective interventions. Therefore, the value of the test-and-reject sampling could be in detecting critical, high-level contamination events that the system may not otherwise adequately control and preventing them from entering the system.

As mentioned earlier, another use for sampling beyond test and reject sampling is microbiological testing used for verification, specifically, process control verification testing (14, 18). A smaller number of samples is taken over time, facilitating analysis of the overall performance of a process. Here, testing is a tool to continuously improve food safety by maintaining or improving the performance of other controls; testing is not used as the primary control (18). This second type of testing could provide value to a leafy green food safety system by identifying unknown or underappreciated pathways of contamination, novel hazards, or other potential risks. For example, pathogen testing environmental data and meteorological data could be used to predict environmental reservoirs of pathogens (36). These types of analyses, particularly if done on data aggregated across producers to represent large portions of the industry, would allow producers and processors to improve their food safety controls to manage new risks across the whole industry.

**(i) Future steps.** Our work represents a variety of contamination spreads, systems, and sampling plans—a total of 147 scenarios. Still, scenarios not addressed in this model are possible. Developing an application programming interface (API) or another user interphase would be beneficial for growers and packers to tune the model to their specific food safety systems and questions. While this model aims to represent a farm-to-customer system for leafy greens, there is still uncertainty about the contamination levels and clustering parameters. As data gaps are filled, the parameters and functions in this model can be updated to deal with this uncertainty, allowing for future analyses with less uncertainty. Future work could also use the model to provide effective sampling plans for a given food safety objective. This would help growers and producers set a testing standard and develop effective sampling plans to meet that standard.

**(ii) Conclusion.** This model was built to assess the efficacy of test-and-reject sampling for farm-to-customer leafy green safety. For this model, the focus was on seven sampling strategies, for three contamination spreads, across seven systems. The results indicate that sampling is less impactful at reducing the endpoint adulterant cells when effective systems-based interventions are in place. The model showed that the sampling plans in this study have limited power to detect contamination levels such as the ones that caused a foodborne disease outbreak in 2018. The model showed that conducting sampling too early in the system may not be as beneficial as sampling closer to a contamination event. Sampling plans should focus on stages at which contamination may be high enough for detection. The stages are preharvest, harvest, and receiving. Other process interventions reduce contamination, resulting in finished product sampling and customer sampling having very low power. However, this study shows that finished product sampling could detect contamination if all interventions were to fail. This study suggests that interventions are effective at reducing incoming contamination. Producers and buyers should focus on implementing good food safety interventions as primary preventive controls. Once interventions are implemented, test-and-reject sampling plans can be used to detect incoming high-level contamination or unappreciated or otherwise uncontrolled sources of contamination.

## MATERIALS AND METHODS

### Model overview.

The model was developed using Python version 3.9.12 (37). It simulated a variety of contamination spreads, food safety interventions, and sampling plans involved in the harvesting, receiving, and processing of leafy greens. A modular process model approach was taken to represent the microbial dynamics of growth, inactivation, mixing, partitioning, removal, and cross-contamination. Figure S1 describes the modules and microbial dynamics, and Table S1 describes the parameters for the model and product flow.

### Product flow.

The initial mass was 100,000 lb of romaine lettuce, chosen to represent a mass reasonably harvested and processed in 1 day by a grower and packer (38). The initial mass was split into 50-lb units to represent the mass as it moves through the system. These 50-lb units were aggregated, mixed, and partitioned as needed to represent processing units such as the production rate, pallet load, and finished product packages. Partitioning and mixing processes were modeled as described by Nauta (39).

### Preharvest contamination.

**FIG 7:**
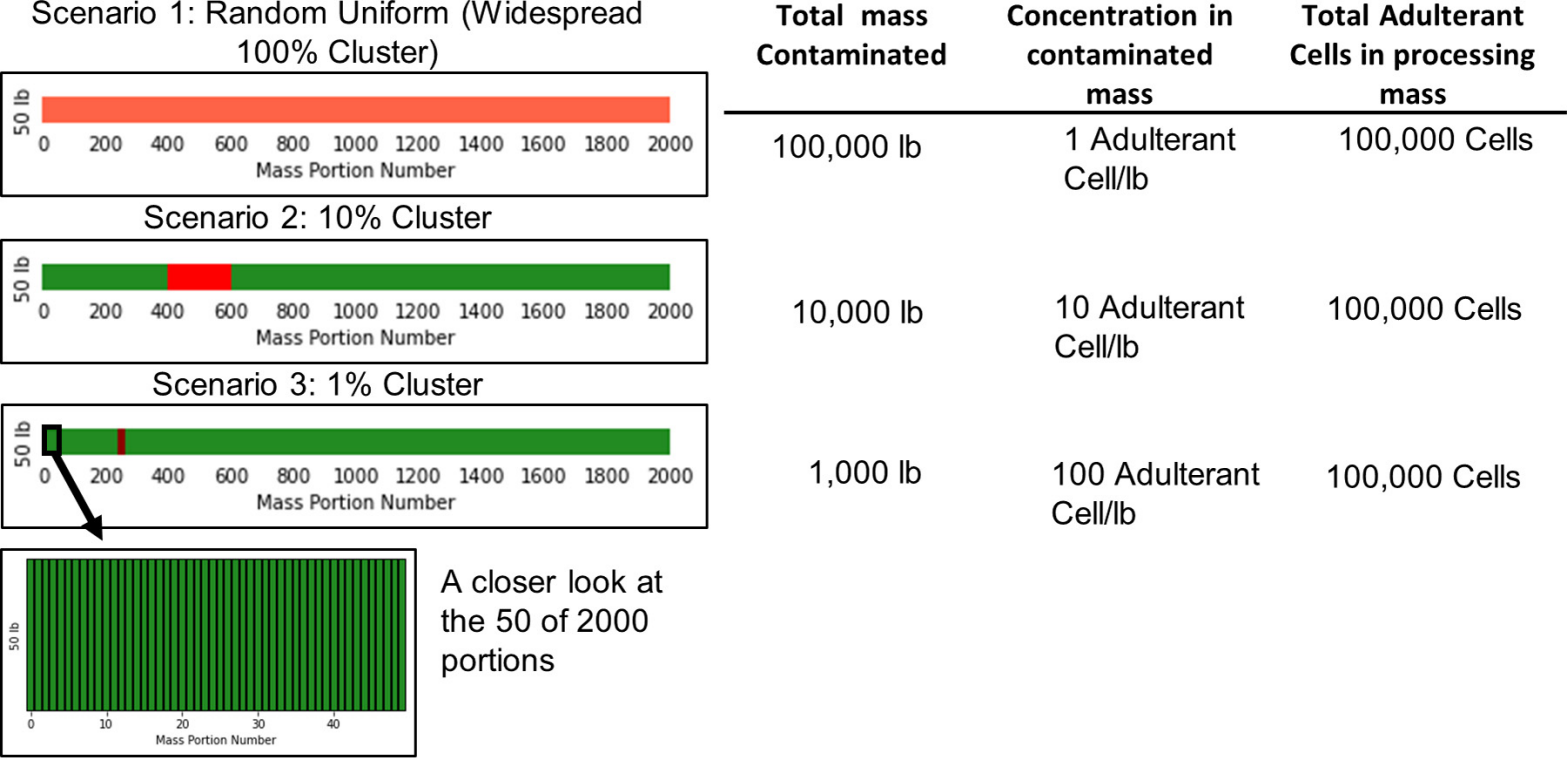
Representation of the 3 contamination scenarios in a 100,000-lb generic mass to be harvested and packed. The light red-shaded area represents the contaminated mass, whereas the darker shading represents a greater concentration of adulterant cells.

Contamination was introduced at preharvest in one of three different patterns (Fig. 7), representing the uncertainty around the actual spread of a contamination event. (i) Random uniform (widespread 100% cluster) contamination: this contamination spread covered the whole mass to be harvested and processed, representing an event such as a field irrigated with contaminated water (10, 32) or rainfall that splashed contaminated soil onto the leafy green leaves, leading to the widespread contamination of the field (10). (ii) Large-cluster contamination: 10% (10,000 lb) of the product was contaminated with an adulterant concentration of 10 CFU/lb. This larger cluster represented a contamination event due to run-off of cattle feces from adjacent farms, contamination from dust containing the adulterant, or other field activities that may have led to contamination in the form of a large cluster (11, 40). (iii) Small cluster contamination: 1% (1,000 lb) of the total mass was highly contaminated (100 CFU/lb). A small-cluster contamination event represented contamination due to animal intrusion or fecal contamination from wild animals, such as bird droppings directly contaminating the leaves or fecal pellets deposited in the field that could be transferred to the leaves due to irrigation or rainfall splash (26, 28, 41).

The total adulterant cells in the process model were fixed at 100,000 cells to provide consistent hazard pressure for empirical scenarios. The 100,000 adulterant cells were distributed over the 100,000-lb mass in patterns dependent on the contamination scenario (random uniform: widespread 100% cluster, large 10% cluster, small 1% cluster). The overall contamination level of the mass was 1 adulterant cell/lb based on reverse engineering performed by the 2020 United Fresh workgroup of the 2018 E. coli O157:H7 romaine lettuce outbreak (42), where the average contamination that likely led to the outbreak was determined to be 0.81 CFU/lb. The goal was to realistically represent a rare event that actual food safety sampling failed to detect, with contamination levels high enough to result in a foodborne disease outbreak. Sporadic background-level contamination was applied to the system at 636 adulterant cells distributed randomly and uniformly across the entire mass of the product; this sporadic contamination level was consistent with the simulated low background contamination in leafy green fields as assumed by Quintanilla Portillo et al. (24).

### Sampling model.

The calculation for the presence of STEC in an individual grab sample was calculated as in Jongenburger et al. (34) following the low-level heterogeneous contamination assumption since it allowed for obtaining the probability of detection of each grab:

(1) $$P_{\mathrm{detect}}=1-e^{-C\cdot M_{\mathrm{GrabSample}}}$$

where *P_detect_* is the probability of detection, *C* is the concentration of the adulterant (adulterant cells/g) in the sampling unit (50-lb unit), and *M_GrabSample_* is the mass of the individual grab sample (g).

Once *P_detect_* was obtained, the presence or absence of the adulterant in the given sample was calculated by checking if a random number between 0 and 1 drawn from a uniform distribution was less than *P_detect_*, meaning the adulterant is present in the grab sample and detected; otherwise, the adulterant is absent from the grab sample and not detected. At the end of each sampling process, if any of the grabs detected an adulterant cell, the product was rejected as part of an International Commission on Microbial Specification for Foods (ICMSF) 2-class attribute sampling plan (22).

### Growth, survival, and die-off models.

Growth and survival were modeled during the transportation and storage of the product. The primary growth model used for this study was a three-phase linear model as proposed by Buchanan et al. (43). The specific growth rate was modeled with a square root model as proposed by Ratkowsky et al. (44). The lag time was calculated as a function of temperature using the power law equation obtained from Mishra et al. (45). It was adapted to represent partial lag consumption under dynamic temperature conditions. When the temperature was under 5°C a log-linear survival model for the adulterant was used as proposed by McKellar et al. (46). The in-field die-off model was adapted from Belias et al. (35), using parameters for E. coli in lettuce; the log-linear parameters were implemented into the model. The combined growth, survival, and die-off models, equations, and parameters are described in detail in File S1.

### Processing steps.

Processing consisted of 6 processing modules: (i) preliminary spray wash, (ii) shredding, (iii) conveyor belt transportation, (iv) flume washing, (v) shaker table, and (vi) dewatering centrifuge as described by Pérez Rodríguez et al. (47). Inactivation and cross-contamination were the primary microbial dynamics during processing.

For the preliminary spray wash, the product underwent a microbial reduction as an effect of spray washing as described by Mokhtari et al. (29). Microbial contaminants were washed off, and the spray wash water was free from contaminants. The reduction of the spray wash was modeled to be between 1.10 and 1.46 log CFU as described by Pahariya et al. (48). Shredding, the conveyor belt, the shaker table, and the dewatering centrifuge represented product-to-surface cross-contamination steps. The approach discussed by Hoelzer et al. (49) and Mokhtari and Van Doren (50) was used to represent cross-contamination between two objects caused by a tactile even, where the total adulterant cells in a 50-lb unit of the total mass were cross-contaminated to the cells on the processing equipment. Specific equations for both the spray wash and cross-contamination are found in File S1.

### Washing.

Flume tank washing was adapted from the model developed by Munther et al. (51). Additional data and parameters were obtained from other studies to fully develop the washing system (29, 38, 52, 53). The dynamic chlorine wash evaluated changes in free chlorine (FC) inside the flume tank as a factor of chemical oxygen demand (COD) (52). The product wash rate was 100 lb/min, which determines the level of organic matter entering the flume wash. The developed system followed the dosing scheme shown by Luo et al. (52), where sodium hypochlorite was added to the flume tank in 12-min intervals. These dosing periods allowed the FC levels to rise back to desired levels. The inactivation and cross-contamination achieved were simulated for the product entering the wash. Equations and parameters for washing dynamics are found in File S1. The code repository contains information on the washing step adaptation and validation. See the “Data Availability” section.

### Processing line sanitation.

The sanitation of four processing equipment surfaces (conveyor belt, shredder, shaker table, and dewatering centrifuge) was simulated as an intervention strategy. The contamination in the processing line was updated for every processing step as every 50-lb portion of the product was processed. Sanitation was modeled as a function of three parameters to represent sanitation at the following steps: (i) sanitation compliance, (ii) sanitation frequency, and (iii) sanitation reduction, where the values for these three parameters are uncertain values randomized on every iteration as shown in Table S1, where sanitation compliance is described as the probability of sanitation occurring, sanitation frequency is the pounds of product that are processed before the processing line is sanitized, and sanitation reduction refers to the log reduction on adulterant cells achieved by sanitation. If the sanitation step occurred, then the new contamination of the processing surface was calculated by applying the corresponding reduction to the processing line as indicated by Mokhtari et al. (29).

### Scenario analysis development.

A scenario analysis was developed, as shown in Fig. 1. The scenario analysis consists of three different contamination spreads, seven systems, and seven sampling plans. The combination of the different factors yielded a total of 147 scenario combinations.

### Production scenarios and interventions.

Five food safety interventions were adapted to represent interventions in a farm-to-customer system. The interventions were preharvest holding, precooling, prewash, chlorinated wash, and processing line sanitation. A detailed description of the interventions can be found in Table 3.

**TABLE 3:** Interventions used to represent good food safety practices

Intervention name	Description
Holding time	The product is harvested 2–8 days after the contamination event, in contrast to the no-intervention system conditions, where the product can be harvested 0–8 days after the contamination event.
Precooling	At receiving, the product is rapidly cooled to 3–5°C; the effect of precooling reduces the temp quickly, potentially preventing the growth of adulterants that may have been exposed to elevated temp during transportation from the farm to the facility.
Spray prewash	As the first step of the production process, the produce is sprayed with a sanitizing solution that is predicted to reduce the microbial load with variable efficacy between (1.1 and 1.46 log adulterant cells).
Chlorinated wash	Chlorinated wash is applied to produce after shredding; this reduces the degree of cross-contamination and reduces adulterants. One of two wash systems is selected if washing is selected as an intervention: (i) the state-of-the-art washing system that constantly maintains FC levels inside the flume tank at 10 ppm or (ii) the washing system with a 2-min dosing period every 12 min.
Processing line sanitation	The processing facility surfaces are washed every 2,500, 5,000, or 7,500 lb of production, with variable compliance (0, 0.25, 0.50, 0.75, 1) and variable efficacy (−1, −2, −3, −4 log cells).

The goal of modeling different systems was to represent the wide range of uncertainty regarding adherence to food safety practices in different operations. Seven systems were generated to address the wide range of uncertainty: an all-intervention system, where all five interventions are applied, a no-intervention system, where no food safety interventions are applied, and five in-between systems, where one of the five food safety interventions was removed at a time. The five additional systems are no holding, no washing, no prewash, no sanitation, and no precooling. This evaluates the benefit of conducting product sampling when a specific food safety intervention fails.

### Sampling scenarios.

Seven separate sampling plans were simulated at different system stages.

1. Preharvest 4 days (PHS 4D): sampling occurred 4 days before harvest.
2. Preharvest 4 h (PHS 4H): sampling occurred 4 hours before harvest.
3. Preharvest intense (PHS Int): sampling occurred immediately before harvest.
4. Harvest sampling (HS): sampling occurred at harvest.
5. Receiving sampling (RS): sampling occurred after temporary storage at the facility.
6. Finished product sampling (FPS): sampling was performed as the shredded product was packed into 5-lb bags.
7. Customer sampling (CS): simulated sampling occurred after transportation from a processing facility to a retail or food service customer.

The sampling plans were designed to match a 1,500-g total composite sample mass and 60 total grabs (Table 1). These sampling plan characteristics were selected to observe guidelines from the International Commission on Microbial Specification for Foods (ICMSF) sampling plan stringency (cases), in this case, ICMSF’s case 15 for severe hazards for which growth may happen (22). The sampling plans also adhered to recommendations from Western Growers’ (WG) Appendix C, which guides growers and processors on preharvest product testing as specified in the Leafy Greens Marketing Agreement (LGMA) approved guidelines (54). These documents recommend that 60 total individual 25-g grabs be tested, resulting in a 1,500-g composite mass. All sampling adhered to the 60 grabs, 1,500-g composite recommendations, except for preharvest sampling intense (PHS Int). For PHS Int, the composite sample mass and the number of grabs were increased 4-fold to observe recommendations made by WG’s Appendix C under the intensified sampling recommendations. WG recommends that for intensified sampling, the maximum sampling area be 1 acre. Since our model simulated a total mass of 100,000 lb, this translated to approximately 4 acres of romaine lettuce harvested (38). Therefore, a total of four 1,500-g 60-grab samples were taken under this scenario.

### Scenario analysis metrics.

A total of 147 combinations were generated for analysis. A total of 10,000 iterations were conducted per combination. The number of iterations needed was computed for the all-intervention and no-intervention systems as a factor of (i) S, the standard deviation of the outputs (endpoint TACs), (ii) E, the desired margin of error (10% of the mean endpoint TACs), and (iii) Z_α/2_ = 1.96, the critical Z value of a normal distribution at a 95% confidence interval (55). More information can be found in “Determination of Total Iterations” in File S1. The following metrics were used to assess the performance of interventions and sampling plans across the scenarios.

Sampling power was a way to measure how well a sampling plan performed at detecting contamination. It is defined as the percentage of times that the sampling plan detected contamination out of the total (*n* = 10,000) number of iterations:

(2) $$\text{Sampling power}=\frac{\text{Model iterations where sampling plan detected pathogen}}{\text{Total iterations}}\times 100$$

The relative efficacy in reducing endpoint total adulterant cells (TACs) from reaching the system’s endpoint was used to quantify how well sampling plans performed in a specific system. The endpoint TAC relative efficacy between the 7 sampling plans was quantified across all 7 systems and compared to each system without sampling as a baseline:

(3) $$\text{Realtive efficacy}=1-\frac{\text{Endpoint}\;\left(\text{TAC}\right)\;,\text{what}-\text{if scenario}}{\text{Endpoint}\;\left(\text{TAC}\right)\;,\;\text{ baseline scenario}}$$

Factor sensitivity analysis (FS) was used to compare interventions and sampling plans. Factor sensitivity is the log reduction between endpoint TACs from each scenario and the system with no food safety interventions or sampling plans (no interventions). The greater the absolute FS, the greater the effect that a specific scenario or condition had on total consumer reduction of endpoint TACs (56, 57):

(4) $$\text{FS =log}\frac{\text{Output (intervention or sampling plan)}}{\text{Output }\;\text{(baseline no}-\text{intervention or sampling plan)}}$$

The partial rank correlation coefficient (PRCC) sensitivity analysis was done as described by Marino et al. (58). The analysis of the outputs was performed in R version 3.6.1 with the function PRCC from the package “sensitivity.” The sensitivity analysis was performed in a system where interventions were turned on, the all-intervention system. The model’s sensitivity was used to determine the system’s most influential parameters for final adulterant cells.

### Data availability.

The code for the process model and the analysis can be found on GitHub (https://github.com/foodsafetylab/Farm-to-Consumer-LG-Sim). Validation documents and a descriptive file with basic instructions on the model files are included in the repository.
