# Supplementary material for: Leafy Green Farm-to-Customer Process Model Predicts Product Testing Is Most Effective at Detecting Contamination When Conducted Early in the System before Effective Interventions
Source: Appl Environ Microbiol. 2023 Apr 26;89(5):e00347-23. doi: 10.1128/aem.00347-23 (PMC10231246; doi:10.1128/aem.00347-23)
Supplement: Supplemental file 1 — Supplemental material. Download aem.00347-23-s0001.docx, DOCX file, 0.9 MB [file aem.00347-23-s0001.docx]

# TECHNICAL SUPPLEMENTAL MATERIALS

## SUPPLEMENTAL METHODS

### The multinomial distribution used to represent contamination event:

To preserve the adulterant cell unit as a whole number, microbial dynamics are a function of binomial processes. To contaminate the field the total adulterant cells were split into the contaminated field partitions by using a multinomial distribution. Contaminated portions of the field were calculated by using a multinomial distribution, following the partitioning concept as shown by Nauta (1):

$\vec{C} \sim\mathrm{Multinomial}(C_{T}, \left( \frac{1}{{CP}_{T}} \right)*{CP}_{T}),$ (1)

where $\vec{C}$ is a vector of adulterant cells, the length of the vector is defined by the number of contaminated partitions dependent on the contamination spread? $C_{T}$ are the total adulterant cells that will be distributed among the contaminated partitions. ${CP}_{T}$ is the total number of contaminated partitions, The second term of the multinomial process is a vector of probabilities of length ${CP}_{T}$ that adds up to 1

### Sampling Plan

Sampling was performed as a factor of three variables: composite sample mass (g), number of grabs (#), and sampling unit (“lot or field”, “sublot”, “pallets”, “finished packages”). The sample mass is the total sample mass taken in the specific sampling unit. The number of grabs is the number of individual grab samples that are taken withing a sampling unit. A sampling unit depends upon the stage of the process where sampling occurs. For example, at preharvest, the sampling units can be the entire lot or a sublot, at receiving sampling, the units can include a “lot or field”, “sublot”, and a “pallet” as the sampling unit. Sampling was performed using simple random sampling with replacement, to simulate completely random sampling of the sampling unit.

The rejection rule for sampling was conducted by obtaining the results from each grab, The sampling process is repeated for every grab sample. The ${Presence}_{y/n}$ results for every grab are recorded into vector $\vec{{Presence}_{y/n}}$. At the end of the sampling process if the sum of numbers in a vector $\vec{{Presence}_{y/n}}$ is greater than 0, the adulterant is present in the composite sample and the lot is rejected, this concept is shown below:

${Reject}_{y/n} = ifelse(\sum_{1}^{n} \vec{{Presence}_{y/n}} > 0, Reject, Accept$) (2)

Where ${Reject}_{y/n}$ is the decision to reject or accept the product based on the results of the sampling unit. $\vec{{Presence}_{y/n}}$ is a vector containing the presence/absence results for each grab sample.

### Growth and Survival Models

The equation to calculate the length of the lag phase was the following:

$t_{L} =7,544*T^{-3.11}, (hours)$ (3)

Where $t_{L}$ is the length of the lag phase (hours), $T$ is the temperature (°C).

Because of temperature changes during transportation and refrigerated storage, there were circumstances where the lag phase was not fully consumed at a given temperature for a given amount of time. Therefore, the remaining lag time had to be calculated at subsequent new temperatures (2). For these circumstances, the portion of lag phase consumed at a given temperature was calculated as:

${PorC}_{L} = \sum_{i}^{n} {t_{i T}}/{t_{L-iT}}$ (4)

Where ${PorC}_{L}$ is the portion of the lag phase consumed (between 0 and 1),$t_{i T}$ is the total time that the product spent in the lag phase at a given temperature (hours). $t_{L-iT}$ is the length of the lag phase at a given temperature (hours). This step can be repeated multiple times if the lag phase is not consumed after multiple temperature changes. Once the ${PorC}_{L}$ accumulated to 1, the lag phase is elapsed, and the exponential phase begins.

Growth during the exponential phase was calculated based using the square root model as proposed by Ratkowsky et al. (3). Where the growth rate and the total growth were calculated as follows:

$R = {(b*(T-T_{min}))}^{2/2.303}, (log (adulterant cells)/h)$ (5)

$\Delta N = R*t, (log adulterant cells)$ (6)

where $R$ is the growth rate at a given temperature. $b$ is the temperature coefficient. $T$ is the temperature (°C). $T_{min}$ is the notional minimum growth temperature (°C). $\Delta N$ is the bacteria population change (log adulterant cells). $t$ is the total growth time (hours).

For the stationary phase, the population change was assumed to be zero, and the number of adulterant cells remained constant.

A survival model was applied when temperatures were below 5 C°. Survival was assumed to follow a log-linear model as specified by McKellar et al. (4). Where the survival rate k, was lognormally distributed with a mean and standard deviation of 0.013 (0.01) log CFU/g with a 0.001 shift.

To represent growth or survival in the system. A Poisson equation was used to represent the fate of individual cells in the overall bacterial population.

$N_{t} =\sum_{i=1}^{N_{0}} Poisson (N_{0}, {10}^{\Delta N}) , (adulterant cells)$ (7)

Where$N_{t}$ is the new population of cells. $N_{0}$ is the initial cell population (cells). *∆*$N$ is the expected population change in log CFU.

## Processing Cross-Contamination and Sanitation processes

The reduction equation for the preliminary spray wash was quantified as follows:

The reduction equation was quantified across the product as follows:

$N =\sum_{i=1}^{N_{0}} Poisson (N_{0}, {10}^{-E_{sw}}), (adulterant cells)$ (8)

Where $N$ is the total number of adulterant cells remaining in the specific partition after spray washing. $-E_{sw}$ is the spray wash efficacy. $N_{0}$ is the initial number of adulterant cells in the partition before the spray wash.

Cross-contamination between product and processing equipment was quantified as follows:

${Tr}_{PS}= \mathrm{Binomial}(N_{0P}, P_{TrPS})$, ($adulterant cells$) (9)

${Tr}_{SP}= \mathrm{Binomial} (N_{0S}, P_{TrSP})$, ($adulterant cells$) (10)

$N_{P}= N_{0P} -{Tr}_{PS} + {Tr}_{SP}$, ($adulterant cells$) (11)

$N_{S}= N_{0S} -{Tr}_{SP} + {Tr}_{PS}$, ($adulterant cells$) (12)

Where ${Tr}_{PS}$ is the total transfer from the product to the surface (adulterant cells), ${Tr}_{SP}$ is the transfer from the surface to the product, $N_{0P}$ is the initial number of adulterant cells in the Product (adulterant cells), $N_{0S}$ is the initial number of adulterant cells on the surface. $P_{TrPS}$ is the transfer coefficient between the Product and the Surface, and$P_{TrSP}$ is the transfer coefficient between the surface and the product (0<$P_{Tr}$<1). $N_{P}$ and $N_{S}$ are the updated contamination in the Product and the Surface.

### Dynamic Wash process

$\frac{d (COD)}{dt} = k_{0}$ (13)

$\frac{d (FC)}{dt} = {-\lambda}_{FC} \cdot FC - \beta_{FC} \cdot FC \cdot COD + \sum_{k=1}^{N} r_{k} \cdot\chi[k_{T,}k_{T + T_{0},}]$ (14)

$\frac{d (X_{W})}{dt} = \beta_{WS}- \beta_{LW} \cdot X_{W}\cdot\frac{L}{V} - \alpha\cdot X_{W} \cdot FC$ (15)

$\frac{d (X_{L})}{dt} ={(X}_{L}\cdot{10}^{(0.214\cdot ln(FC)+0.220)}) + \beta_{LW}\cdot X_{W}$ (16)

$\beta_{WS} = \sigma\cdot(1 -X_{s})\cdot\frac{\theta N}{V}$ (17)

The first equation represents the change in chemical oxygen demand (COD). This is a function of the amount of organic mattering entering the tank. Results obtained from Luo et al, at wash rate of 100 lb./ min.

The second equation is the change in free chlorine inside the flume tank.

The third equation is the change of adulterant cells in the wash water. Where the first term $\beta_{WS}$ represents the rate of adulterant cells entering the water. The second term represents the contact an attachment of the adulterant cells to the produce, and the third term represents the inactivation of the inoculated adulterant cells due to FC.

The fourth equation was modified and adapted from both Munther and Madamba, since the model uses lettuce as the vehicle and receiving produce, the fourth equation from Munther et al was modified (5). The first term represents the inactivation of adulterant cells in the contaminated product inside the flume tank, this equation was adapted from Mandamba et al (6). The second term from the equation represents the adulterant cells binding from the water to the lettuce inside the wash tank.

The fifth equation represents the rate of adulterant cells entering the wash water. As explained by Munther et al. $X_{s}$ values were obtained from Mokhtari et al.

Where COD is the chemical oxygen demand (mg/L), $k_{0}$ rate of change for COD per unit time (mg/ L min), FC is the level of free chlorine in the wash water (mg/L), $\lambda_{FC}$ is the natural decay rate of free chlorine in water (1/min), $\beta_{FC}$ is the depletion rate of free chlorine in the wash water (L/mg min). $r_{k}$is the added rate of free chlorine at the desired dose (mg/ mL min^2^). $\chi$ is an indicator function, where it is 1 if the time is withing the dosing period time interval $[k_{T,}k_{T + T_{0},}]$ , and 0 if the dosing period is off. $X_{W}$ is the concentration of adulterant cells in the wash water (Cells /ml). $\beta_{LW}$ is the adulterant cells binding rate to pieces of lettuce (mL/ g min). $\beta_{WS}$ is the rate of adulterant cells entering the wash water (Cells / ml min). $L$ is the amount of lettuce in the wash tank at a given time (g). $V$ is the volume of the flume tank (mL). $\alpha$ is the inactivation rate for suspended adulterant cells via free chlorine (L / mg min). $X_{L}$ is the concentration of adulterant cells of the lettuce inside the wash tank. $\sigma$ is the concentration of adulterant cells in the lettuce before entering the wash tank (Cells /g). $X_{s}$ is the fraction of adulterant cells that remain in the lettuce pieces during washing. $\theta N$ is the rate of spinach coming into the wash tank (g / min).

## Processing line sanitation

Reduction to the processing line as indicated by Mokhtari et al (7):

$N_{S}= \mathrm{binomial} (N_{0S}, {10}^{SE})$, (adulterant cells) (18)

Where $SE$ is the sanitation efficacy for the surface of the processing equipment, $N_{S}$ and $N_{0S}$ are the contamination levels on the surface, after and before sanitation, respectively.

### Determination of total Iterations

The number of iterations of the model were calculated according to Winston (8) which proposes that the number of iterations, n, should be a factor of (i) S, standard deviation of the outputs, (ii) E, desired margin of error, and (iii) Z_α/2_ = 1.96, the critical Z value of a normal distribution at a 95% confidence interval.

$n = {[Za/z* S/E]}^{2}$ (19)

The All-Intervention and the No-Intervention system for 10,000 iterations were used to assess if the number of simulated iterations was sufficient. The main output of the model, Total Adulterant Cells (TAC) at the customer was used to calculate the output standard deviation, S = 2.52 and 5,600 TACs for the All-Intervention and No-Intervention systems, respectively. The desired margin of error E was computed to be 10% from the mean, E= 0.59 * 0.1 = 0.059, and E= 1,585 * 0.1 = 159 for the All-Intervention and No-Intervention systems, respectively. When plugged into equation 19, we obtained 6,920 and 4,888 iterations for the All-Intervention and No-Intervention systems, respectively. So, running 10,000 iterations will allow us to be 95% confident that the mean output will be accurate within ± 0.059 TACs, and ± 160 TACs for the All-Intervention and No-Intervention systems, respectively.

### Partial Rank Correlation Sensitivity Analysis

Following the factor sensitivity analysis, a partial rank correlation sensitivity analysis (PRC) was conducted for all the model variable inputs over their parametrized range. The sensitivity of the model was used to determine the most influential parameters for final adulterant cells in the system. The sensitivity analysis was performed in a system where interventions were turned on, the All-Intervention system. The partial rank correlation coefficient was used as the metric for the analysis as described by Marino et al. (9) The analysis of the outputs was performed in R version 3.6.1 and with the function PRCC from the package “sensitivity”.

### Validation of sampling 1D setup vs 2D setup.

In a previous study conducted by this group (10), a simulation of preharvest sampling was built to predict the probability of rejection of a generic one-acre field given sample mass (g), number of grabs (#), and contamination spread, under simple random sampling conditions. This model was validated against published data by (11).To validate the model and ensure that this linear mass setup did not introduce bias, two contamination scenarios were compared. (i) The first contamination scenario was a field randomly contaminated, with: 1 CFU/10kg (-4 log (CFUs)/g), 1 CFU /kg (-3 log (CFUs)/g), 1 CFU/ 100g (-2 log (CFUs)/g) and 1 CFU/10g (-1 log (CFUs)/g). The sample size was variable with sizes of 180, 360, 900, 1800, 3600 g, and a variable number of 3g grabs, 60, 120, 300, 600, 1200. (ii) The second contamination scenario was for a highly clustered contamination, where 0.3% of the field was contaminated at a contamination level of 1000 CFU/g (3 log (CFU)/g). This scenario also was simulated as a factor of variable sample mass: 180, 360, 900, 1800, 3600 g, and a variable number of grabs: 60, 120, 300, 600, 1200. To consider the model validated all the sampling conditions from the current study must fall within the 2.5^th^ – 97.5^th^ percentile from the validation study.

## SUPPLEMENTAL RESULTS

### Preharvest sampling was successfully validated.

The current study was validated against a previously validated study by Quintanilla-Portillo et al. The validation of the model was done for two contamination scenarios: (i) uniform contamination of the field, for 4 different contamination levels, and (ii) Point source, highly clustered contamination at a single contamination level. **Figure S3** displays the comparison between the validated study and the results from this study. For the model to be considered validated the means of the current study must fall within the 2.5^th^ -97.5^th^ percentile of the already validated study. For the point source contamination, while this model slightly underpredicts the median probability of acceptance compared to the validation, it does fall withing the 2.5^th^ and 97^th^ percentile, so the model is considered validated. For the uniform contamination, the mean of the current study falls withing the 2.5^th^ and 97^th^ percentile of the validation for all four different contamination levels, demonstrating that the model can predict probability of acceptance under uniform contamination conditions. As the number of individual grabs increase, the probability of acceptance decreases, and as the contamination levels decrease, the probability of acceptance increases. The model is validated for both uniform and point source contamination, indicating that the current model is capable of accurately determining sampling plan power as a function of sample mass (g), and number of grabs (#) for different contamination levels and spreads.

## TABLES AND FIGURES:

TABLE S1: Parameter table for generalized leafy green model.

|  | Name | Description | Value | | Unit | Main Reference | Secondary Reference or justification |
| --- | --- | --- | --- | --- | --- | --- | --- |
| *Initial Mass Setup* | | | | | | | |
|  | Total_Weight | Weight of mass to be harvested and processed | 100,000 | Lb. | | User Inputs.  Otherwise baseline 100,000 lb. field.(12) | Assuming the amount of lettuce processed by a processing facility in a day.  ~150 bins of 650 lb. each. This is approximately 100 lb. of produce (13) |
|  | Slot_weight | Sublot Weight | 10,000 | Lb. | | User Input  Otherwise, 10,000 lb. | 1/10^th^ of the total mass considered a sublot |
|  | Pallet_Weight | Weight of a pallet of product | 4,000 | Lb. | | User Input  Otherwise, 4,000 lb. |  |
|  | Pack_Final_Weight | Weight of bag of chopped romaine lettuce | 5 | Lb. | | Industry Standards. | Assuming finished product is a 5lb. bag of chopped romaine lettuce for foodservice use. |
|  | Case_Weight | Weight of case with units f romaine. | 20 | Lb. | | Industry Standard | 4 X 5lb. bags in the case. 20 lb. is typical case weight for the industry. |
| *Contamination Scenarios* | | | | | | | |
|  | Hazard_lvl | Hazard Level | 100,000 | Adulterant cells or 1 Adulterant Cell/lb. | | User Input | Based on analysis for 2018 Romaine lettuce outbreaks. |
|  | Cluster_Size | Size of cluster | 1.100,000  2.1,000  3.10,000 | Lb.  Numbers represent contamination scenarios | | User Input | Representation of different contamination spread that may occur in primary productions |
|  | Cluster_No | Number of clusters in field | 1 | Cluster | | User Input |  |
| *Time and Temperature* | | | | | | | |
|  | Time_CE_H | Time between Irrigation - Harvest | Triangular (0,4,8)  **Holding:** Triangular (2,4,8) | days | | (14) | From FDA Leafy green risk assessment. Recommends 2-8 days of holding time post irrigation. |
|  | Temperature_ColdStorage | Temperature cold storage processing facility | Normal (5.1, 0.27) | °C | | (15) |  |
|  | Time_ColdStorage | Time Cold storage at facility | 1 | Hours | | Assumed |  |
|  | Temperature_H_Receiving | Temperature from Harvest to Receiving | Uniform (15,17) | °C | | (16) |  |
|  | Time_H_Receiving | Time between Harvest and Receiving | 1 | Hours | |  |  |
|  | Temperature Receiving Storage | Temperature at Receiving, before processing | Triangular (0 ,8 ,14) | °C | | (6, 17) |  |
|  | Time Receiving Storage | Time of temporary storage at receiving | Uniform (0,1) | Hours | | (18) |  |
|  | Transportation Temperature | Temperature during transportation | Triangular (0 ,4 ,6) | °C | | (17) |  |
|  | Transportation Time | Transportation time between facility and foodservice/retail operation | Uniform (31.17, 89.92) | Hours | | (19) |  |
|  | Temperature CS Storage | Storage Time at Retail/ Foodservice | Triangular (0 ,8 ,14) | °C | | (6, 17) |  |
|  | Time CS Storage | Storage Time at Retail/ Foodservice | Uniform (24,72) | Hours | | (17, 18) |  |
| *Die-off Model E. coli* | | | | | | | |
|  | Linear Die-off | Die-off rates segment 1 | Normal (-0.77, 0.21) | Log (Adulterant cells)/ day | | (20) | Log-linear model was chosen instead of the segmented model because it resulted in less variable results. |
| *Growth Model above 5°C* | | | | | | | |
|  | b | Temperature coefficient | 0.023 |  | | (4, 21) |  |
|  | T_min_ | Theoretical minimum temperature | 1.2 | °C | | (4, 21) |  |
|  | Lag_Time | Equation to calculate lag time as a factor of temperature | *7,544*T^-3.011^* | Hours | | (4, 21) |  |
|  | Nmax | Population Max | 7 | Log (CFU)/g | | (14) |  |
| *Survival Model below 5°C* | | | | | | | |
|  | k | Survival rate is temperature is lower to 5°C | Triangular (0.0035,0.013,0.04) /2.303 | Log (CFU)/g h | | (4) |  |
| *Processing* | | | | | | | |
| Transfer between food and processing equipment | | | | | | | |
|  | P_TrSh-P_ | Shredder to lettuce | Triangular (0.16,0.2,0.28) | % /100 | | (7) | Other study (22) evaluated the cross-contamination during processing. It evaluated contamination rates from Produce to each individual equipment, but it evaluated transfer from equipment to produce. Therefore, for the purposes of our model we went with a model that would allow us to evaluate transfer at each individual step. |
|  | P_TrP-Sh_ | Lettuce to Shredder | Triangular (0,0.0025,0.0053) | % /100 | | (7) |  |
|  | P_TrCv-P_ | Conveyor Belt to Lettuce | Triangular (0.15,0.18,0.22) | % /100 | | (7) |  |
|  | P_TrP-Cv_ | Lettuce to Conveyor Belt | Triangular (0,0.0062,0.0139) | % /100 | | (7) |  |
|  | P_TrSt-P_ | Shaker Table to Lettuce | Triangular (0.06,0.28,0.30) | % /100 | | (7) |  |
|  | P_TrP-St_ | Lettuce to Shaker Table | Triangular (0,0.0006,0.0038) | % /100 | | (7) |  |
|  | P_TrC-P_ | Centrifuge to Lettuce | Triangular (0.23,0.27,0.31) | % /100 | | (7) |  |
|  | P_TrP-C_ | Lettuce to Centrifuge | Triangular (0,0.0035,0.0159) | % /100 | | (7) |  |
| *Washing* | | | | | | | |
|  | E_sw_ | Spray Wash Efficacy | Uniform (1.1-1.46) | Log (Adulterant Cells) | | (23) |  |
|  | Wash_rate | Rate at which lettuce enters the flume tank | 100  45 | Lb. /min  Kg/min | | (24) |  |
| Chlorine Levels over time | | | | | | | |
|  | r1 | FC addition rate of dose 1 | **C Wash:** 12.75  No C Wash: 0 | mg/(ml(min)^2^) | | (5) |  |
|  | r2 | FC addition rate of dose 2 | **C Wash:** 7.47  No C Wash: 0 | mg/(ml(min)^2^) | | (5) |  |
|  | r3 | FC addition rate of dose 3-n | **C Wash:** 5.56  No C Wash: 0 | mg/(ml(min)^2^) | | (5) |  |
|  | Ro | Chlorine dosing period | 12 | min | | (5) |  |
|  | Ro_0_ | Chlorine dosing period duration | 2 | min | | (5) |  |
|  | K_0_ | Free Chlorine demand per minute | 32.3 | mg/ (L min) | | (5) |  |
|  | Λ_c_ | Natural decay of FC | 1.7*10^-3^ | 1/Min | | (5) |  |
|  | Β _FC_ | Depletion rate of FC in wash water | 5.38*10^-4^ | L/ (mg min) | | (5) |  |
| *Washing Action* | | | | | | | |
|  | Β_LW_ | Pathogen binding rate to lettuce from water | 0.38 | Ml/g | | (5) |  |
|  | α | Inactivation rate of pathogen via FC | 0.75 | L/ (mg min) | | (5) |  |
|  | V | Volume of flume tank | 3200 | L | | (24) |  |
|  | Wash Time | Reciprocal of average wash time | 2.3 | 1/min | | (5) |  |
| *Sanitation and Sanitation compliance parameters* | | | | | | | |
|  | Compliance | Sanitation compliance | Discrete (0,0.25,0.5,0.75,1.0 | - | | (7) | Sanitation compliance chosen as a discrete distribution by original study. Sensitivity of the parameters will be analyzed |
|  | Frequency | Frequency | Discrete (2,500, 5,000, 7,500) | Lb. | | Assumed |  |
|  | Efficiency |  | Discrete (1 ,2 ,3 ,4) |  | | (7) | Effect will be evaluated in sensitivity analysis. |

TABLE S2: Sampling Plan Power Results. Supplement to **Figure 5** in the manuscript

| **Step** | No Interventions | All Interventions | No Washing | No Prewash | No Holding | No Sanitation | No Precooling |
| --- | --- | --- | --- | --- | --- | --- | --- |
| *Uniform Random (Widespread 100% Cluster) Contamination* | | | | | | | |
| Preharvest 4 days (PHS 4D) | 23.1% | 29.6% | 29.6% | 29.3% | 22.4% | 29.2% | 29.0% |
| Preharvest 4 hours (PHS 4H) | 7.7% | 1.7% | 1.5% | 1.7% | 7.3% | 1.5% | 1.5% |
| Preharvest Sampling Intense (PHS Int) | 15.1% | 4.3% | 4.4% | 4.7% | 15.5% | 4.5% | 4.5% |
| Harvest Sampling (HS) | 6.2% | 1.4% | 1.1% | 1.3% | 6.4% | 1.2% | 1.4% |
| Receiving Sampling (RS) | 7.0% | 1.6% | 1.5% | 1.5% | 7.4% | 1.4% | 1.6% |
| Finished product Sampling (FPS) | 7.3% | 0.0% | 0.1% | 0.1% | 0.0% | 0.0% | 0.0% |
| End Consumer Sampling (CS) | 4.0% | 0.0% | 0.0% | 0.0% | 0.0% | 0.0% | 0.0% |
| *10% Contamination* | | | | | | | |
| Preharvest 4 days (PHS 4D) | 21.4% | 28.4% | 28.0% | 28.2% | 21.3% | 28.2% | 29.5% |
| Preharvest 4 hours (PHS 4H) | 6.7% | 1.5% | 1.4% | 1.5% | 7.2% | 1.4% | 1.5% |
| Preharvest Sampling Intense (PHS Int) | 15.1% | 4.7% | 4.9% | 4.5% | 15.1% | 4.2% | 4.0% |
| Harvest Sampling (HS) | 5.9% | 1.4% | 1.2% | 1.3% | 6.4% | 1.3% | 1.1% |
| Receiving Sampling (RS) | 7.3% | 1.5% | 1.4% | 1.6% | 6.7% | 1.4% | 1.6% |
| Finished product Sampling (FPS) | 7.3% | 0.0% | 0.1% | 0.1% | 0.0% | 0.0% | 0.0% |
| End Consumer Sampling (CS) | 4.0% | 0.0% | 0.0% | 0.0% | 0.0% | 0.0% | 0.0% |
| *1% Contamination* | | | | | | | |
| Preharvest 4 days (PHS 4D) | 14.1% | 18.0% | 18.0% | 17.9% | 14.1% | 17.6% | 18.1% |
| Preharvest 4 hours (PHS 4H) | 5.4% | 1.4% | 1.3% | 1.7% | 5.3% | 1.3% | 1.6% |
| Preharvest Sampling Intense (PHS Int) | 13.4% | 4.1% | 4.2% | 4.4% | 13.4% | 4.3% | 4.4% |
| Harvest Sampling (HS) | 4.4% | 1.3% | 1.3% | 1.2% | 4.7% | 1.2% | 1.1% |
| Receiving Sampling (RS) | 5.5% | 1.5% | 1.2% | 1.4% | 5.4% | 1.4% | 1.4% |
| Finished product Sampling (FPS) | 7.0% | 0.0% | 0.1% | 0.1% | 0.0% | 0.0% | 0.0% |
| End Consumer Sampling (CS) | 3.7% | 0.0% | 0.0% | 0.1% | 0.0% | 0.0% | 0.0% |

TABLE S3: Relative Efficacy of sampling plans. This is the data presented in **Figure 6** in the Manuscript

| Random Uniform (Widespread 100% Cluster) Contamination | | | | | | | | | | | | | | |
| --- | --- | --- | --- | --- | --- | --- | --- | --- | --- | --- | --- | --- | --- | --- |
|  | Baseline No-Intervention | Relative Efficacy | Baseline All-Intervention | Relative Efficacy | No Holding | Relative Efficacy | No Precool | Relative Efficacy | No Prewash | Relative Efficacy | No Wash | Relative Efficacy | No Sanitation | Relative Efficacy |
| No Sampling | 15,474,923 | 0% | 5,802 | 0% | 38,321 | 0% | 6,619 | 0% | 106,097 | 0% | 113,535 | 0% | 5,709 | 0% |
| PHS 4D | 14,679,401 | 5% | 4,798 | 17% | 36,858 | 4% | 5,282 | 20% | 88,635 | 16% | 100,767 | 11% | 4,813 | 16% |
| PHS 4H | 7,162,502 | 54% | 5,135 | 11% | 19,740 | 48% | 5,881 | 11% | 94,270 | 11% | 101,862 | 10% | 5,032 | 12% |
| PHS Int | 3,614,542 | 77% | 4,258 | 27% | 8,290 | 78% | 4,502 | 32% | 75,910 | 28% | 85,119 | 25% | 3,841 | 33% |
| HS | 7,999,065 | 48% | 5,383 | 7% | 19,833 | 48% | 5,816 | 12% | 92,465 | 13% | 102,419 | 10% | 5,033 | 12% |
| RS | 7,396,794 | 52% | 5,247 | 10% | 19,080 | 50% | 5,953 | 10% | 93,043 | 12% | 101,191 | 11% | 4,913 | 14% |
| FPS | 7,147,289 | 54% | 5,826 | 0% | 38,303 | 0% | 6,596 | 0% | 105,411 | 1% | 112,461 | 1% | 5,709 | 0% |
| CS | 9,551,045 | 38% | 5,804 | 0% | 38,261 | 0% | 6,632 | 0% | 105,279 | 1% | 112,756 | 1% | 5,704 | 0% |
| 10% Cluster Contamination | | | | | | | | | | | | | | |
|  | Baseline No-Intervention | Relative Efficacy | Baseline All-Intervention | Relative Efficacy | No Holding | Relative Efficacy | No Precool | Relative Efficacy | No Prewash | Relative Efficacy | No Wash | Relative Efficacy | No Sanitation | Relative Efficacy |
| No Sampling | 15,495,257 | 0% | 6,143 | 0% | 40,415 | 0% | 7,113 | 0% | 112,491 | 0% | 123,798 | 0% | 6,070 | 0% |
| PHS 4D | 15,091,965 | 3% | 4,773 | 22% | 37,304 | 8% | 5,386 | 24% | 87,778 | 22% | 100,852 | 19% | 4,579 | 25% |
| PHS 4H | 7,630,794 | 51% | 5,616 | 9% | 20,243 | 50% | 6,235 | 12% | 101,182 | 10% | 109,726 | 11% | 5,571 | 8% |
| PHS Int | 3,752,442 | 76% | 4,542 | 26% | 8,844 | 78% | 4,500 | 37% | 75,275 | 33% | 88,614 | 28% | 4,184 | 31% |
| HS | 8,254,350 | 47% | 5,680 | 8% | 20,380 | 50% | 6,474 | 9% | 101,273 | 10% | 111,967 | 10% | 5,311 | 13% |
| RS | 7,844,791 | 49% | 5,340 | 13% | 19,761 | 51% | 6,216 | 13% | 100,644 | 11% | 109,331 | 12% | 5,333 | 12% |
| FPS | 7,553,763 | 51% | 6,057 | 1% | 40,139 | 1% | 6,578 | 8% | 109,040 | 3% | 123,545 | 0% | 6,093 | 0% |
| CS | 9,936,448 | 36% | 6,143 | 0% | 39,402 | 3% | 6,998 | 2% | 111,696 | 1% | 122,991 | 1% | 5,993 | 1% |
| 1% Cluster Contamination | | | | | | | | | | | | | | |
|  | Baseline No-Intervention | Relative Efficacy | Baseline All-Intervention | Relative Efficacy | No Holding | Relative Efficacy | No Precool | Relative Efficacy | No Prewash | Relative Efficacy | No Wash | Relative Efficacy | No Sanitation | Relative Efficacy |
| No Sampling | 15,571,633 | 0% | 5,757 | 0% | 38,908 | 0% | 6,161 | 0% | 102,793 | 0% | 120,943 | 0% | 5,850 | 0% |
| PHS 4D | 15,118,971 | 3% | 4,914 | 15% | 37,127 | 5% | 5,869 | 5% | 93,557 | 9% | 108,978 | 10% | 4,812 | 18% |
| PHS 4H | 11,054,119 | 29% | 5,264 | 9% | 26,908 | 31% | 5,652 | 8% | 96,411 | 6% | 109,112 | 10% | 5,233 | 11% |
| PHS Int | 4,931,492 | 68% | 4,361 | 24% | 12,459 | 68% | 4,304 | 30% | 75,459 | 27% | 84,937 | 30% | 4,180 | 29% |
| HS | 10,903,805 | 30% | 5,356 | 7% | 26,790 | 31% | 5,757 | 7% | 97,279 | 5% | 113,151 | 6% | 5,438 | 7% |
| RS | 11,180,254 | 28% | 5,196 | 10% | 26,109 | 33% | 5,832 | 5% | 94,548 | 8% | 109,130 | 10% | 5,211 | 11% |
| FPS | 8,266,344 | 47% | 5,775 | 0% | 38,904 | 0% | 6,216 | -1% | 101,932 | 1% | 119,553 | 1% | 5,833 | 0% |
| CS | 10,565,098 | 32% | 5,759 | 0% | 38,826 | 0% | 6,205 | -1% | 102,678 | 0% | 120,691 | 0% | 5,766 | 1% |

TABLE S4: Prevalence of contaminated portions at endpoint. This data was not presented in the manuscript. This table shows that sampling plans have limited efficacy at reducing the prevalence of contaminated packages when good intervention are in place, compared to when no at effective interventions are in place.

| Random Uniform (Widespread 100% Cluster) Contamination | | | | | | | | | | | | | | |
| --- | --- | --- | --- | --- | --- | --- | --- | --- | --- | --- | --- | --- | --- | --- |
|  | Baseline No-Intervention | Relative Efficacy | Baseline All-Intervention | Relative Efficacy | No Holding | Relative Efficacy | No Precool | Relative Efficacy | No Prewash | Relative Efficacy | No Wash | Relative Efficacy | No Sanitation | Relative Efficacy |
| No Sampling | 3.65% | 0% | 0.0020% | 0% | 0.0134% | 0% | 0.0019% | 0% | 0.0370% | 0% | 0.0397% | 0% | 0.0020% | 0% |
| PHS 4D | 3.50% | 4% | 0.0017% | 17% | 0.0128% | 4% | 0.0015% | 19% | 0.0308% | 17% | 0.0353% | 11% | 0.0017% | 15% |
| PHS 4H | 1.87% | 49% | 0.0018% | 9% | 0.0068% | 49% | 0.0017% | 11% | 0.0328% | 11% | 0.0357% | 10% | 0.0017% | 12% |
| PHS Int | 0.98% | 73% | 0.0015% | 27% | 0.0029% | 78% | 0.0013% | 31% | 0.0265% | 28% | 0.0296% | 26% | 0.0013% | 32% |
| HS | 2.08% | 43% | 0.0019% | 8% | 0.0070% | 47% | 0.0017% | 11% | 0.0324% | 12% | 0.0357% | 10% | 0.0018% | 11% |
| RS | 1.93% | 47% | 0.0018% | 10% | 0.0067% | 50% | 0.0017% | 11% | 0.0325% | 12% | 0.0353% | 11% | 0.0017% | 13% |
| FPS | 1.90% | 48% | 0.0020% | 0% | 0.0134% | 0% | 0.0019% | 0% | 0.0368% | 0% | 0.0393% | 1% | 0.0020% | 0% |
| CS | 2.56% | 30% | 0.0020% | 0% | 0.0134% | 0% | 0.0019% | 0% | 0.0368% | 1% | 0.0395% | 1% | 0.0020% | 0% |
| 10% Cluster Contamination | | | | | | | | | | | | | | |
|  | Baseline No-Intervention | Relative Efficacy | Baseline All-Intervention | Relative Efficacy | No Holding | Relative Efficacy | No Precool | Relative Efficacy | No Prewash | Relative Efficacy | No Wash | Relative Efficacy | No Sanitation | Relative Efficacy |
| No Sampling | 1.85% | 0% | 0.0021% | 0% | 0.0138% | 0% | 0.0020% | 0% | 0.0379% | 0% | 0.0426% | 0% | 0.0021% | 0% |
| PHS 4D** | 1.76% | 5% | 0.0017% | 22% | 0.0128% | 7% | 0.0015% | 24% | 0.0302% | 20% | 0.0345% | 19% | 0.0016% | 24% |
| PHS 4H | 1.21% | 35% | 0.0019% | 9% | 0.0069% | 50% | 0.0018% | 12% | 0.0343% | 9% | 0.0376% | 12% | 0.0019% | 10% |
| PHS Int | 0.78% | 58% | 0.0015% | 27% | 0.0030% | 78% | 0.0013% | 35% | 0.0259% | 32% | 0.0306% | 28% | 0.0014% | 33% |
| HS | 1.31% | 29% | 0.0020% | 7% | 0.0071% | 49% | 0.0018% | 9% | 0.0342% | 10% | 0.0385% | 10% | 0.0018% | 13% |
| RS | 1.24% | 33% | 0.0019% | 11% | 0.0067% | 51% | 0.0018% | 12% | 0.0341% | 10% | 0.0375% | 12% | 0.0018% | 13% |
| FPS | 1.23% | 34% | 0.0021% | 1% | 0.0136% | 1% | 0.0019% | 5% | 0.0369% | 3% | 0.0424% | 0% | 0.0021% | -1% |
| CS | 1.51% | 19% | 0.0022% | -2% | 0.0135% | 2% | 0.0020% | 1% | 0.0377% | 1% | 0.0422% | 1% | 0.0020% | 5% |
| 1% Cluster Contamination | | | | | | | | | | | | | | |
|  | Baseline No-Intervention | Relative Efficacy | Baseline All-Intervention | Relative Efficacy | No Holding | Relative Efficacy | No Precool | Relative Efficacy | No Prewash | Relative Efficacy | No Wash | Relative Efficacy | No Sanitation | Relative Efficacy |
| No Sampling | 1.10% | 0% | 0.0020% | 0% | 0.0130% | 0% | 0.0018% | 0% | 0.0342% | 0% | 0.0404% | 0% | 0.0020% | 0% |
| PHS 4D | 1.05% | 5% | 0.0017% | 14% | 0.0125% | 4% | 0.0017% | 7% | 0.0310% | 9% | 0.0363% | 10% | 0.0017% | 15% |
| PHS 4H | 0.89% | 19% | 0.0018% | 9% | 0.0090% | 31% | 0.0016% | 9% | 0.0319% | 7% | 0.0365% | 10% | 0.0018% | 10% |
| PHS Int | 0.60% | 45% | 0.0015% | 25% | 0.0043% | 67% | 0.0013% | 30% | 0.0252% | 26% | 0.0287% | 29% | 0.0014% | 29% |
| HS | 0.92% | 17% | 0.0019% | 6% | 0.0091% | 30% | 0.0017% | 7% | 0.0324% | 5% | 0.0378% | 6% | 0.0018% | 9% |
| RS | 0.90% | 18% | 0.0018% | 11% | 0.0088% | 32% | 0.0017% | 5% | 0.0313% | 9% | 0.0367% | 9% | 0.0019% | 8% |
| FPS | 0.83% | 25% | 0.0020% | 0% | 0.0131% | 0% | 0.0018% | -2% | 0.0340% | 1% | 0.0401% | 1% | 0.0020% | 2% |
| CS | 0.94% | 14% | 0.0020% | 0% | 0.0130% | 0% | 0.0018% | 0% | 0.0342% | 0% | 0.0404% | 0% | 0.0020% | 3% |


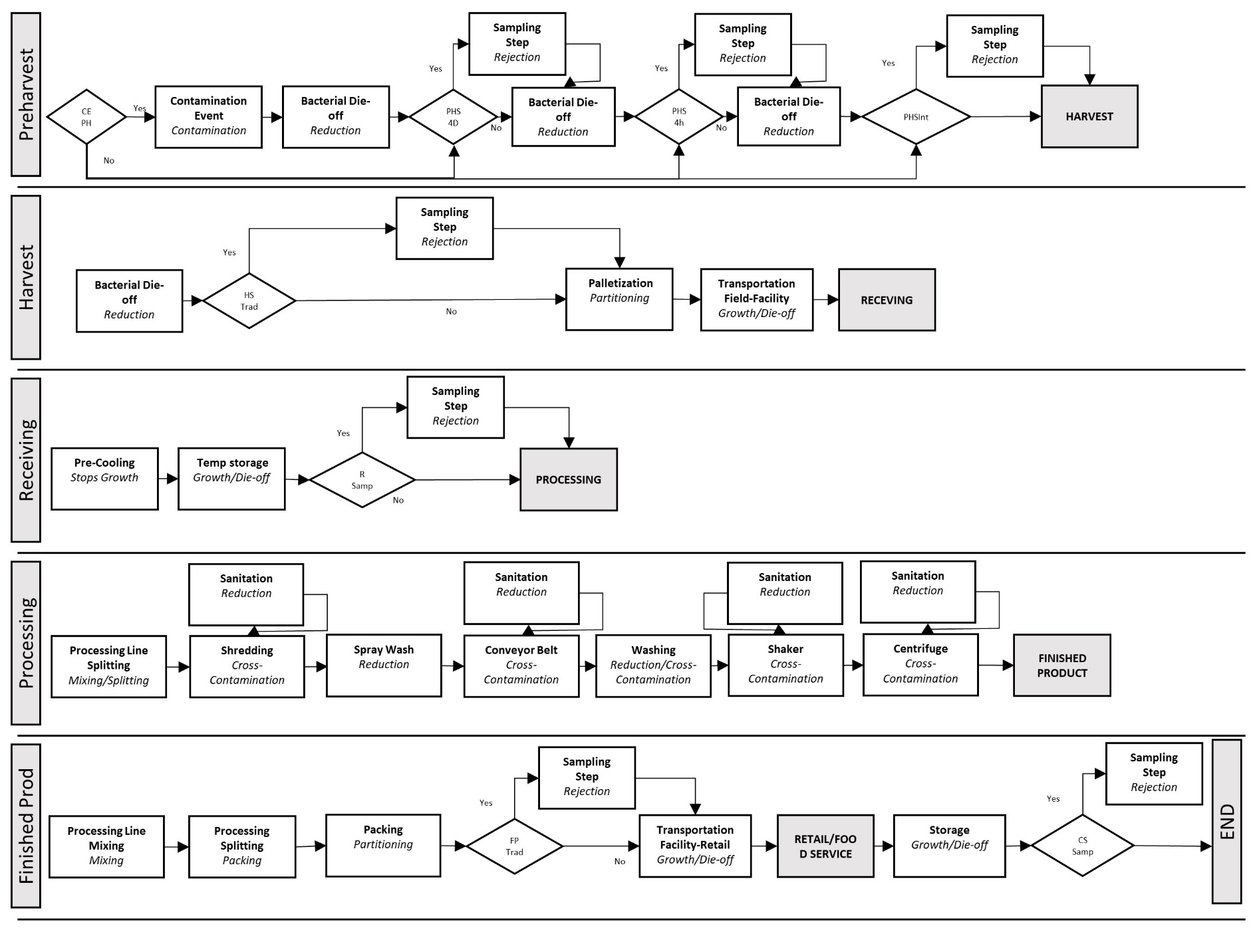


FIGURE S1. Model steps and framework. Each row represents a module for the model. Each module contains a set of decision and steps that eventually lead to the following module. Microbial dynamics are italicized, whereas the main model steps are in bold.


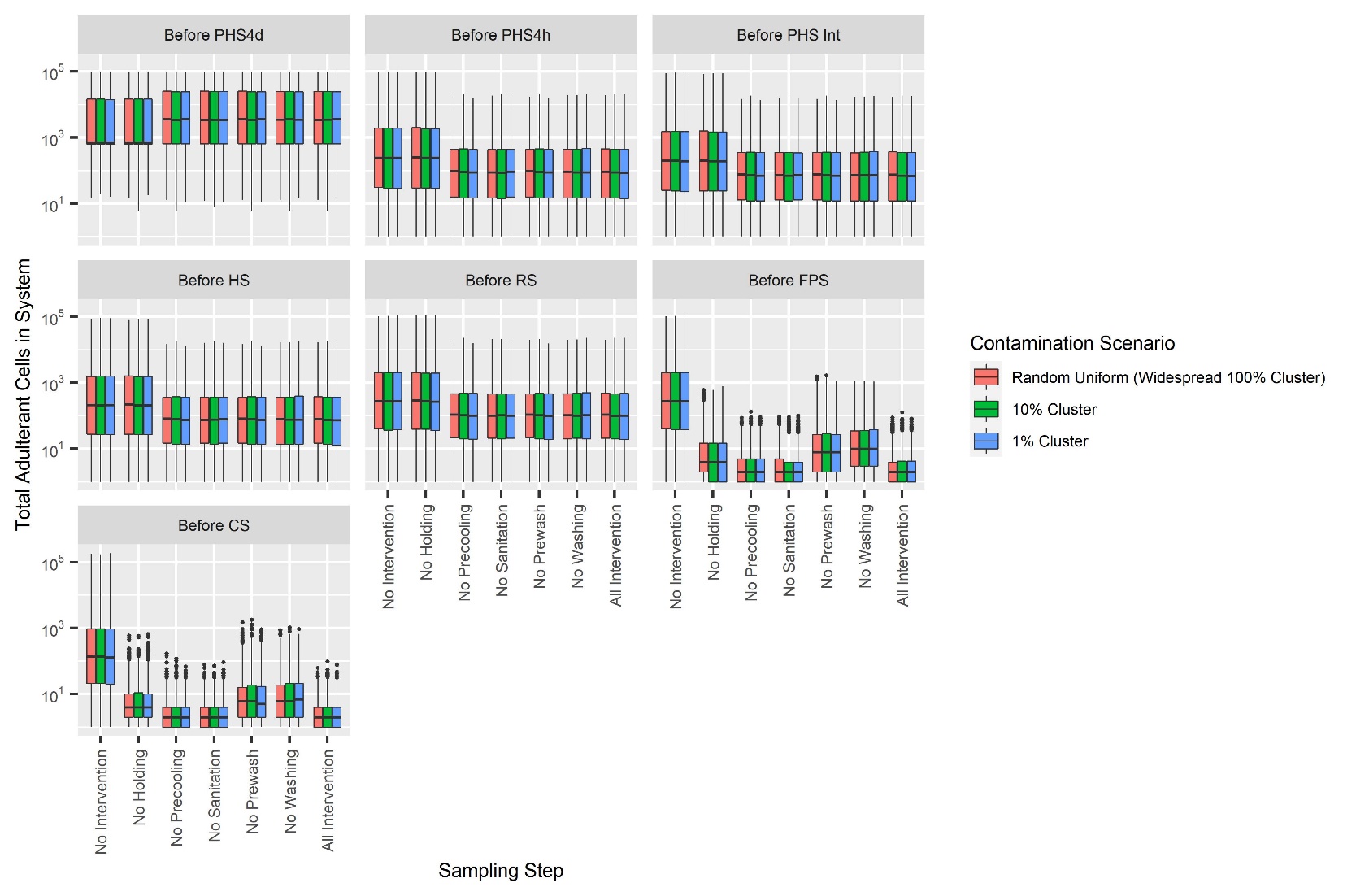


FIGURE S2: Total Adulterant Cells (TAC) in the system before each sampling plan. Contaminations are stratified for each of the 7 processing systems.


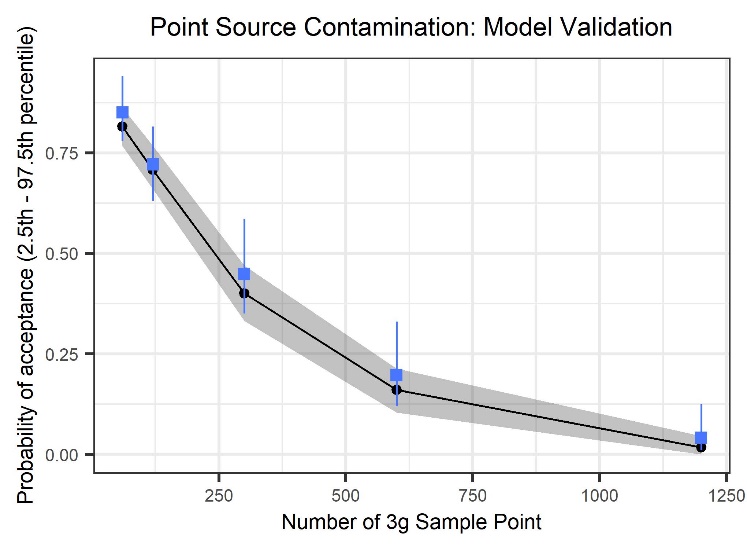

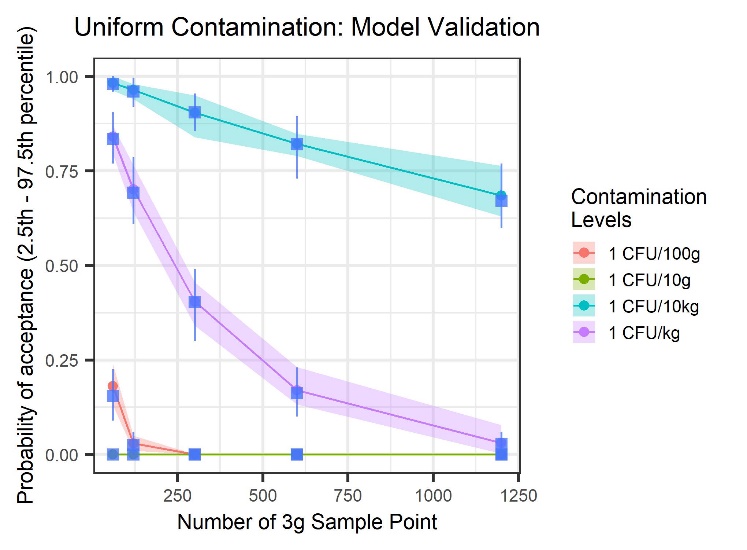


FIGURE S3: Validation of this model against validated model by Quintanilla Portillo et al (25). Left: Validation of sampling performance under point source contamination. Blue boxes and error bars represent the median and 2.5th - 97.5th percentile from previous model (25). Black line represents the mean from current study with shaded area the 2.5th - 97.5th percentile. All simulated performance means fall within the 2.5th - 97.5th percentile of previous project. Therefore, model sampling performance is validated for point source contamination. Right: Validation of sampling plan under uniform contamination. Same graph construction. All simulated performance means fall within the 2.5th - 97.5th percentile of previous project. Therefore, model sampling performance is validated for uniform contamination.

## REFERENCES

1. Nauta MJ. 2007. The Modular Process Risk Model (MPRM): a Structured Approach to Food Chain Exposure Assessment, p 99-136. *In* Schaffner Donald W, Doyle MP (ed), Microbial Risk Analysis of Foods doi:<https://doi.org/10.1128/9781555815752.ch4>. ASM Press, Washington, D.C.

2. Stasiewicz MJ, Martin N, Laue S, Gröhn YT, Boor KJ, Wiedmann M. 2014. Responding to Bioterror Concerns by Increasing Milk Pasteurization Temperature Would Increase Estimated Annual Deaths from Listeriosis. Journal of Food Protection 77:696-705.

3. Ratkowsky DA, Olley J, McMeekin TA, Ball A. 1982. Relationship between temperature and growth rate of bacterial cultures. Journal of Bacteriology 149:1-5.

4. Mckellar RC, Delaquis P. 2011. Development of a dynamic growth–death model for *Escherichia coli* O157:H7 in minimally processed leafy green vegetables. International Journal of Food Microbiology 151:7-14.

5. Munther D, Luo Y, Wu J, Magpantay FMG, Srinivasan P. 2015. A mathematical model for pathogen cross-contamination dynamics during produce wash. Food Microbiology 51:101-107.

6. Madamba T, Moreira RG, Castell-Perez E, Banerjee A, da Silva D. 2022. Agent‐based simulation of cross‐contamination of *Escherichia coli* O157:H7 On lettuce during processing with temperature fluctuations during storage in a produce facility. Part 1: Model development Journal of Food Process Engineering 45.

7. Mokhtari A, Oryang D, Chen Y, Pouillot R, Van Doren J. 2018. A Mathematical Model for Pathogen Cross-Contamination Dynamics during the Postharvest Processing of Leafy Greens. Risk Analysis 38:1718-1737.

8. Winston WL. 2000. Simulation Modeling Using @Risk. Duxbury Press.

9. Marino S, Hogue IB, Ray CJ, Kirschner DE. 2008. A methodology for performing global uncertainty and sensitivity analysis in systems biology. Journal of Theoretical Biology 254:178-196.

10. Quintanilla Portillo J, Cheng X, Belias AM, Weller DL, Wiedmann M, Stasiewicz MJ. A Validated Preharvest Sampling Simulation Shows that Sampling Plans with a Larger Number of Randomly Located Samples Perform Better than Typical Sampling Plans in Detecting Representative Point-Source and Widespread Hazards in Leafy Green Fields. Applied and Environmental Microbiology 0:e01015-22.

11. Xu A, Buchanan RL. 2019. Evaluation of sampling methods for the detection of pathogenic bacteria on pre-harvest leafy greens. Food Microbiology 77:137-145.

12. Brennan J, Schaffner DW, Stasiewicz MJ, Tamborello L, Wilhelmsen E, Rosen J. 2021. Would my sampling plan have detected contamination levels that resulted in an outbreak? A Thought Experiment. Accessed

13. Mokhtari A, Pang H, Santillana Farakos S, Davidson GR, Williams EN, Van Doren JM. 2021. Evaluation of Potential Impacts of Free Chlorine during Washing of Fresh‐Cut Leafy Greens on *Escherichia coli* O157:H7 Cross-Contamination and Risk of Illness. Risk Analysis 42:966-988.

14. Pang H, Lambertini E, Buchanan RL, Schaffner DW, Pradhan AK. 2017. Quantitative Microbial Risk Assessment for *Escherichia coli* O157:H7 in Fresh-Cut Lettuce. Journal of Food Protection 80:302-311.

15. Tromp SO, Rijgersberg H, Franz E. 2010. Quantitative Microbial Risk Assessment for Escherichia coli O157:H7, Salmonella enterica, and Listeria monocytogenes in Leafy Green Vegetables Consumed at Salad Bars, Based on Modeling Supply Chain Logistics. Journal of Food Protection 73:1830-1840.

16. Koseki S, Isobe S. 2005. Prediction of pathogen growth on iceberg lettuce under real temperature history during distribution from farm to table. International Journal of Food Microbiology 104:239-248.

17. Zeng W, Vorst K, Brown W, Marks BP, Jeong S, Pérez-Rodríguez F, Ryser ET. 2014. Growth of Escherichia coli O157:H7 and Listeria monocytogenes in Packaged Fresh-Cut Romaine Mix at Fluctuating Temperatures during Commercial Transport, Retail Storage, and Display. Journal of Food Protection 77:197-206.

18. Ohio State University Extension. 2017. Current Sanitation Practices for Leafy Green Vegetables For Processors, Retailers and Consumers. <https://ohioline.osu.edu/factsheet/aex-261>. Accessed 01-13.

19. Brown W, Ryser E, Gorman L, Steinmaus S, Vorst K. 2016. Transit temperatures experienced by fresh-cut leafy greens during cross-country shipment. Food Control 61:146-155.

20. Belias AM, Sbodio A, Truchado P, Weller D, Pinzon J, Skots M, Allende A, Munther D, Suslow T, Wiedmann M, Ivanek R. 2020. Effect of Weather on the Die-Off of *Escherichia coli* and Attenuated *Salmonella enterica* Serovar Typhimurium on Preharvest Leafy Greens following Irrigation with Contaminated Water. Applied and Environmental Microbiology 86.

21. Mishra A, Guo M, Buchanan RL, Schaffner DW, Pradhan AK. 2017. Prediction of *Escherichia coli* O157:H7, *Salmonella,* and *Listeria monocytogenes* Growth in Leafy Greens without Temperature Control. Journal of Food Protection 80:68-73.

22. Pérez Rodríguez F, Campos D, Ryser ET, Buchholz AL, Posada-Izquierdo GD, Marks BP, Zurera G, Todd E. 2011. A mathematical risk model for Escherichia coli O157:H7 cross-contamination of lettuce during processing. Food Microbiology 28:694-701.

23. Pahariya P, Fisher DJ, Choudhary R. 2022. Comparative analyses of sanitizing solutions on microbial reduction and quality of leafy greens. LWT 154:112696.

24. Luo Y, Nou X, Millner P, Zhou B, Shen C, Yang Y, Wu Y, Wang Q, Feng H, Shelton D. 2012. A pilot plant scale evaluation of a new process aid for enhancing chlorine efficacy against pathogen survival and cross-contamination during produce wash. International Journal of Food Microbiology 158:133-139.

25. Stasiewicz MJ. 2021. Simulation analysis of in-field produce sampling for risk-based sampling plan development.
